# Decoding nucleic acid contributions to phase separation and ordering in biomolecular condensates

**DOI:** 10.1093/nar/gkag253

**Published:** 2026-03-30

**Authors:** Daniele Asnicar, Simone Codispoti, Carlo Morasso, Alberta Ferrarini, Giuliano Zanchetta

**Affiliations:** Department of Chemical Sciences, University of Padova, Via Marzolo 1, 35131 Padova, Italy; Department of Medical Biotechnology and Translational Medicine, Università degli Studi di Milano, via F.lli Cervi 93, 20054 Segrate, Italy; Laboratory of Nanomedicine, Istituti Clinici Scientifici Maugeri IRCCS, Via Maugeri 10, 27100 Pavia, Italy; Department of Chemical Sciences, University of Padova, Via Marzolo 1, 35131 Padova, Italy; Department of Medical Biotechnology and Translational Medicine, Università degli Studi di Milano, via F.lli Cervi 93, 20054 Segrate, Italy

## Abstract

Liquid-liquid phase separation (LLPS) is being increasingly recognized as a major organizational principle for proteins and nucleic acids (NAs) in cells, as well as a promising strategy for synthetic biology and biomedical applications. Extensive work has explored the role of protein sequence and properties in the regulation of LLPS. Despite some relevant exceptions, the role of nucleic acids has been much less explored. Here, to fill this gap, we focus on model systems made of oligonucleotides with tuned lengths and degree of hybridization, mixed with a moderately charged, disordered peptide. Combining multiple length scales through experiments and molecular dynamics simulations, we unravel the distinct effects of the properties of NAs on the phase behavior, and we propose a metric for the stability of biocondensates. We also characterize the conditions for the onset of liquid crystalline order in the droplets, and we show that it is associated with a dramatic slowing down of the dynamics of oligonucleotides, while peptides retain high mobility. Our results can be generalized to natural and non-natural NAs of arbitrary structure, providing a guide for the design of synthetic NA-containing coacervates.

## Graphical Abstract

**Graphical Abstract:**
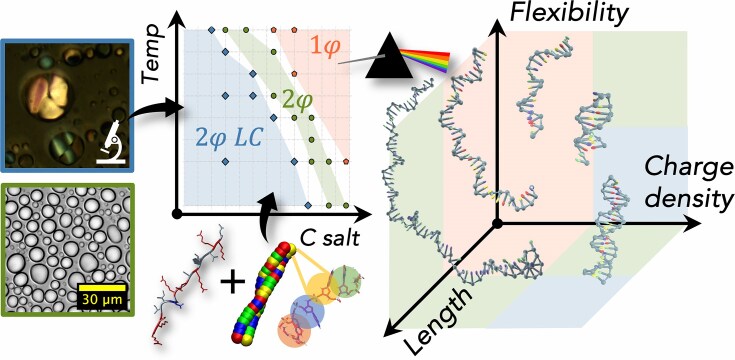

## Introduction

The interactions between proteins and nucleic acids (NAs) are crucial for countless cellular functions, from the assembly and activity of the ribosome [1] to transcription regulation [2]. In particular, NAs can regulate protein solubility and activity through the formation of membraneless organelles, also called biomolecular condensates or coacervates, arising from spontaneous liquid-liquid phase separation (LLPS) of protein–NA complexes [3, 4]. This mechanism, which may have provided enrichment and colocalization in prebiotic times [5, 6], is increasingly recognized as a major principle for cellular organization, from gene expression [2] to DNA repair and response to stress [7, 8], but is also associated with the onset of pathological protein aggregation [9, 10]. Furthermore, LLPS is being investigated as a promising strategy for synthetic biology [11] and drug delivery [12], whose rational design, however, requires robust understanding and fine control [13].

The role of solution conditions (ions, crowders) [14] and of protein sequence and charge patterning in shaping the properties of biomolecular condensates has been widely investigated and is rather well established [3, 9, 15–22]. In contrast, fewer studies have been conducted on the contribution of NAs, often with contradictory observations [4, 10, 23]. Compared to proteins, NAs have less pronounced sequence variability and are characterized by a highly charged backbone, which can overwhelm base composition effects. Overall, purines display a higher propensity for phase separation in the presence of positively charged amino acids (AA) [24] or divalent ions [25–27], which has been correlated to base stacking free energy [24], direct interaction with amino acids [28], and solvent–nucleotide (NT) interactions.

The distinct ability of NAs to base-pairing gives rise to double-stranded (ds) constructs, which are less flexible, have higher charge density, and, by virtue of the lower exposure of the nucleobases, weaker hydrophobic character than single strands (ss). This combination of factors results in a strong effect on coacervation, but it is difficult to unravel each individual contribution [29], also because most studies have been conducted on mixtures of oligonucleotides (ONTs) with highly charged polylysine strands [30–33]. The more flexible single-stranded DNA displays coacervate droplets in a wide range of conditions [34]. Instead, dsDNA often forms solid precipitates which dissolve at high enough ionic strength, with isotropic liquid and liquid crystalline (LC) droplets at intermediate salt concentrations [31, 32] (in the latter case, the term liquid-liquid crystalline phase separation - LLCPS - is used [35, 36]). This general trend agrees with simulation and theory results, showing inhibition of coacervation because of the entropic penalty of aligning neighboring chains [37] and a weaker degree of structural correlation [38, 39] for rigid polyelectrolytes. However, other studies involving portions of the Histone binding protein report conflicting results, with increased stability for the more highly charged dsDNA and guanosine (G) quadruplexes [40, 41], or, on the contrary, enhanced phase separation for ssDNA droplets relative to dsDNA [42]. Even the persistence of the secondary structure inside the condensates is disputed [43, 44].

**Figure 1.**
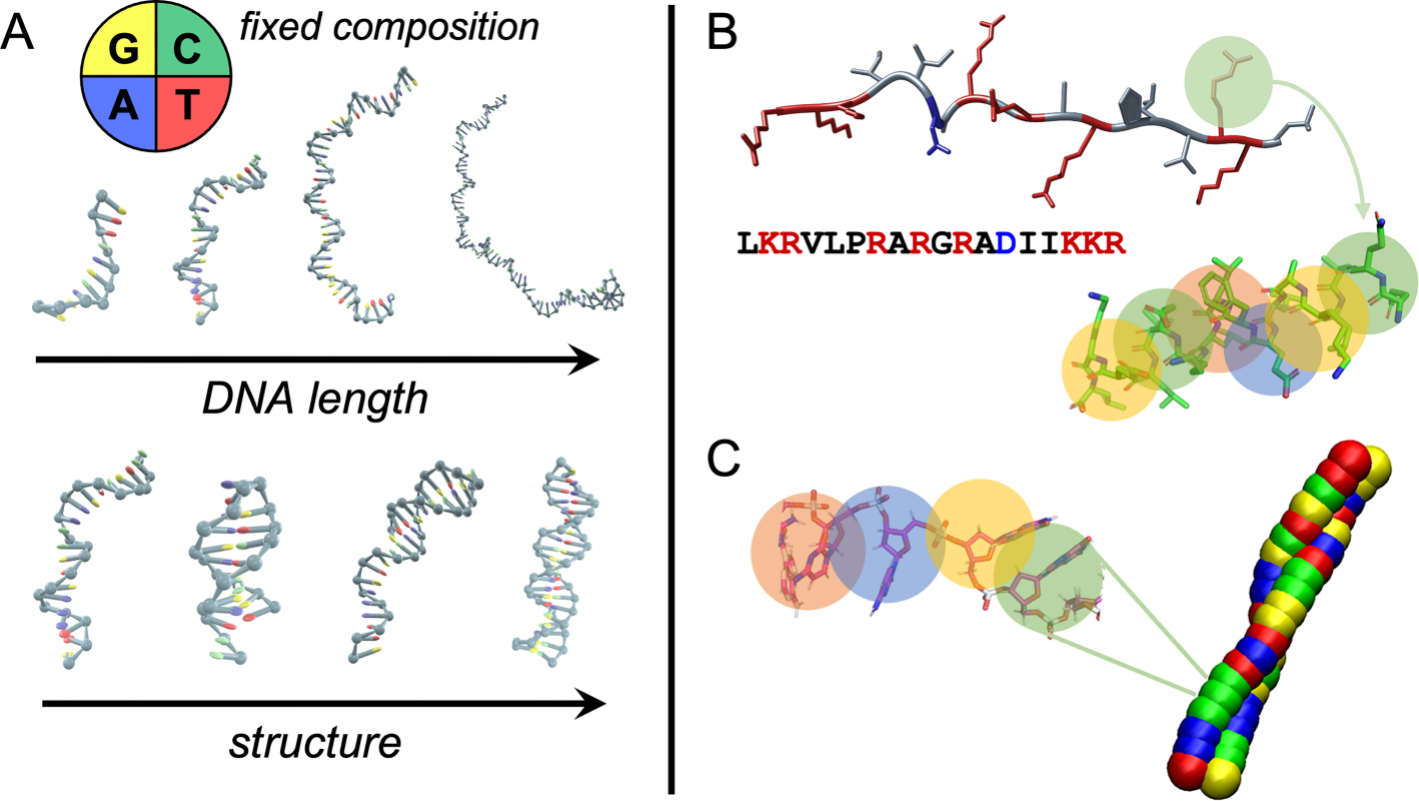
Overview of some DNA oligomers investigated in this study. (**A**) oxDNA sketches of DNA oligomers of balanced NTs composition, including single strands of different lengths, along with fully and partially base-paired strands (see Supplementary Table S2). (**B**) Structure and sequence of the pL22 peptide (neutral amino acids are indicated in black, cationic AAs in red, anionic AAs in blue) and corresponding CG model, where each bead represents an amino acid. (**C**) CG model of dsDNA adopted in MD simulations, with one-bead representation of nucleotides.

Here, we systematically explore the phase behavior of NA oligomers of different lengths and secondary structures, including partially structured systems (Fig. 1A), when mixed with a disordered peptide carrying a moderate positive charge and without pronounced sequence patterns (Fig. 1B). This condition favors a more realistic comparison with naturally occurring proteins, better mimics the NA interactions within biomolecular condensates [30], and allows the differences between NA structures, often leveled out by peptides with a high charge density, to emerge more clearly. Moreover, we confirm the link between DNA structure and condensate stability by assessing the hybridization of the DNA oligomers inside the condensates by means of Raman microscopy. Besides equilibrium properties, the mobility of the molecular species inside the droplet is also estimated from Fluorescence Recovery After Photobleaching (FRAP) experiments.

The experimental investigation is complemented by simulations based on a coarse-grain (CG) model with resolution at the level of a single nucleotide/amino acid and parameters tuned to capture sequence-specific interactions and mechanical properties of the polyelectrolytes [17, 45, 46], which we adapt to include NA hybridization and capture the effect of secondary structure of ONTs. CG models cannot provide an explicit description of phenomena such as solvent reorganization, salt partitioning, or counterion release upon binding [31, 47–49]. Nevertheless, they have been found to capture the main effects of the phase behavior of protein-NA mixtures, greatly contributing to our understanding of LLPS and of the material properties of biomolecular condensates [41, 50, 51]. Here, we apply CG modeling to calculate phase diagrams and to investigate aspects that elude direct experimental observation, such as the interaction network, the degree of order, and the kinetics of biomolecular species inside the coacervates. By independently tuning parameters that cannot be fully disentangled in experiments, we provide support to the mechanistic explanations for the observed behavior. We establish robust quantifiers of flexibility and charge distribution for various ONTs of different lengths and secondary structures and dissect their role in coacervate stability.

We extend the investigation to peptide nucleic acids (PNA) and polyphosphate (polyP), which bear biological and biotechnological interest. In particular, PNAs, with nucleobases attached to a neutral, peptidic backbone, also thanks to their specific pairing to DNA and RNA [52], are a powerful tool for diagnostics, gene editing, and drug delivery [53]. PolyPs, which instead have a similar backbone to DNA and RNA but lack nucleobases, are ancient and ubiquitous inorganic polymers interacting with chromatin [54, 55]. Their propensity to form condensates has recently attracted attention in the context of prebiotic research [56] and modulation of gene expression [57, 58]. Within our framework, we also model and explain the phase behavior of such biomolecular species.

## Materials and methods

### Sequence design

Sequence optimization was performed with the aid of the *NUPACK Python module* [59]. Using the approach detailed in Supplementary Data, we selected the ssDNA-10, ssDNA-20, ssDNA-40, and hp5DNA oligomers. Instead, dsDNA-10, dsDNA-20, dsDNA-40, and hdsDNA were obtained by hybridization of the aforementioned ssDNA sequences with their fully or partially complementary traits (see Supplementary Table S2).

### Molecular dynamics simulations

We extended the CG model developed in Refs. [17, 45, 46], where a bead-spring representation of NAs and peptides is used, with each bead corresponding to an amino acid or a nucleotide in implicit solvent. Electrostatic interactions between the beads are described by the Debye–Hückel potential with a temperature-dependent water permittivity [60]. For short-range, non-bonded interactions, a modified Lennard–Jones potential [61] is used, wherein the attractive contribution is scaled by a so-called hydropathy parameter accounting for the hydrophobicity of the beads.

The chemical and structural specificity of the systems, critical for comparison to experiments, is taken into account through a tailored parametrization of the interactions (details in Supplementary Data and Supplementary Table S1). For amino acids, we adopted the parameters proposed in Ref. [46], with distinct hydropathy values and charges (either 0 or $\pm$*e*) for each residue. Each nucleotide was assigned a charge of $-$*e*, whereas specific hydropathy parameters were recalculated for DNA, following the original procedure [45]; the results indicate a slightly higher hydrophobicity compared to that of their RNA counterparts. To tune the stiffness of NA chains, we added a harmonic bending potential between triplets of consecutive beads in a strand. Finally, to model base-pairing in ds tracts to account for double-stranded structures, we introduced a harmonic stretching potential between beads representing hydrogen-bonded pairs. For PNA, the same DNA model and parameters were adopted, with charges switched off. In the case of polyP, a hydropathy parameter equal to 0 was assumed in view of its very hydrophilic nature, whereas specific geometric constraints and parameters were defined in accordance with its chemical structure.

In simulations, we used a peptide concentration $C^{sim}_{pL22}=120$ mM, significantly higher than the experimental one, namely $C^{exp}_{pL22}=300\, \mu$M, to make the systematic investigation of phase separation easier. For some selected cases, we verified that the composition of the dense phase was not affected.

Phase separation was investigated using the slab method [17]. To build the phase diagrams, several simulations were run at different temperatures and ionic strengths. For specific state points, we performed bulk simulations of larger systems using periodic boundary conditions in the NPT ensemble with zero external pressure [62]. All simulations were carried out using the LAMMPS package [63].

### Quantification of chain flexibility

The flexibility of DNA oligomers was estimated using a normalized Root Mean Square Deviation (here referred as $RMSD_n$) to account for the intrinsic length dependence of the RMSD [64] in short and highly charged systems. For all the standard nucleic acids employed, the RMSD was estimated by performing *oxDNA* CG simulations [65] as detailed in Supplementary Data. Because the time-averaged RMSD scales linearly with the length of the DNA strands (see Supplementary Data, Supplementary Fig. S10), we implemented the simple normalization:


(1) \begin{eqnarray*}
RMSD_n \doteq \frac{RMSD}{\mathcal {N}}
\end{eqnarray*}

where $\mathcal {N}$ is the number of monomers of the ONT, i.e., single nucleotides for ssDNA, base pairs for dsDNA, quartets for G-quadruplexes, and the proper combination for partially hybridized structures (in analogy with the definition of the contour length ${\mathcal {L}}_c$).

### Calculation of the surface electrostatic potential

To calculate the average electrostatic potential generated on the surface of a polyanion by its charge distribution, we used atomistic representations of the systems, with the atomic coordinates, the partial charges, and atomic radii obtained using the APBS-PDB2PQR software suite [66]. The molecular surface was defined by a set of points placed over a uniform grid in a rectangular box containing the molecule. The Coulomb potential $\phi _e$ was evaluated at each point, assuming a relative dielectric constant $\epsilon =2$ inside the polyanions. The average surface potential $\Phi _e$ was then calculated over the grid by discrete summation. The defined $\Phi _e$ bears an intrinsic dependence on the polymer length, arising from the long-range nature of the Coulomb potential and from the combinatorial growth of the grid points where the potential is evaluated. Therefore, we normalize the average surface potential $\Phi _e$ as (see Supplementary Data for detailed explanation):


(2) \begin{eqnarray*}
\widetilde{\Phi }_e \doteq \frac{\Phi _e}{\ln \mathcal {N}}
\end{eqnarray*}

### Oligonucleotides

ssDNA oligomers (see Supplementary Table S2) were purchased from IDT (Integrated DNA Technologies) and used without further purification (standard desalting). Lyophilized oligomers were resuspended in Milli-Q water at $100-500\, \mu$M concentrations and stored at $-20\, ^{\circ }$C.

Hybridized DNA sequences were obtained by mixing 1:1 the sequence of interest with its complementary segment and by performing a slow temperature annealing with a thermoblock.

Purified linear polyphosphate at 200 mM concentration was kindly supplied by RegeneTiss.Inc (Japan) in the form of EX-polyP^®^ with average chain lengths of 20. Stock solutions were subsequently diluted down to $500\, \mu$M and stored at $-20\, ^{\circ }$C.

HPLC-purified PNA, where the negatively charged sugar-phosphate backbone is substituted by a neutral pseudo-peptide skeleton composed of N-(2-aminoethyl)-glycine units, was purchased from Biomers.net GmbH and subsequently dissolved in Milli-Q water at $500\, \mu$M concentration. To aid dissolution, the solution was kept shaking at 600 rpm and at $60\, ^{\circ }$C for several minutes. The concentration of PNA was then verified using a NanoReady Touch micro-volume spectrophotometer. The PNA-10 hybrid was obtained by mixing 1:1 the PNA stock solution with its DNA complement. Before performing a temperature annealing, the solvent conditions were adjusted to 10 mM NaCl, 20 mM Tris–HCl, pH 7.7 and $5\%\, v/v$ acetonitrile. The measured UV absorbance melting curve (see Supplementary Data, Supplementary Fig. S17) confirmed that hybridization took place.

### Peptide

pL22 is an ancestral protein fragment of the large ribosomal subunit of *Thermus thermophilus* (see PDB 4V51). It is a basic peptide of 18 AAs, enriched in ARG and LYS (see Supplementary Table S3) and lacking a well-defined secondary structure in solution, although in the ribosome it adopts a peculiar $\beta$-loop folding as part of a larger domain.

The pL22 lyophilized stock was synthesized by the Spyder Institute using standard protocols for solid-phase peptide synthesis; it was further dissolved in 20 mM Tris–HCl buffer (pH 7.7) at 2 mM concentration and stored at $-20\, ^{\circ }$C.

### Raman microscopy

Raman spectra were acquired using a Raman microspectrometer (InVia Reflex, Renishaw plc, Wotton-under-Edge, UK) equipped with a 532 nm laser line. Raman measurements on coacervates were performed using an excitation laser at 100% power (50 mW nominal value at the source), a 1800 L/mm grating, and a Leica 100× objective. For each sample, a $10\, \mu$L aliquot was sandwiched through a Raman grade $CaF_2$ disc and a glass cover slip, separated by $50\, \mu$m spacers.

For point measurements, spectra from at least five distinct coacervates were acquired, each spectrum recorded as a sum of 5 accumulations of 2 s each. Raman spatial maps were obtained instead by scanning a region of interest comprising one or more condensates and a supernatant portion; Raman spectra were acquired at each point with similar settings as the ones used for point measurements, with spectra collected every $0.5\, \mu$m and an accumulation time of 0.5 s per point.

For the measurement of dried reference samples, a $5\, \mu$L drop of molecules dispersed in water was deposited on the surface of a Raman grade $CaF_2$ disk and left to dry under flow for 20 min. Spectra were acquired using the same settings, and each spectrum was recorded as the sum of ten 3 s accumulations.

For spectra pre-processing and analysis, see Supplementary Data.

### Mobility measurements

Fluorescently labeled ssDNA-20 and dsDNA-20 coacervate samples were prepared for confocal microscopy according to the procedure for phase diagram characterization (see Supplementary Data), but with the addition of TAMRA-labeled pL22 and FAM-labeled DNA added in a 1:200 ratio with respect to the unlabeled molecule concentrations.

Immediately after preparation, samples were sandwiched between two glass BSA-coated coverslips, separated by a silicone gasket of thickness 1.0 mm with a circular aperture of diameter 9 mm (GraceBio-Labs FastWells^TM^ reagent barriers). After the cell was sealed, the samples were allowed to equilibrate for about 2 h at room temperature.

The liquidity of the coacervate samples and the molecular mobility within the condensates were assessed by Fluorescence Recovery After Photobleaching (FRAP), as described in Supplementary Data.

## Results and discussion

To decouple the role of the molecular features of NAs from the sequence composition, we select DNA strands featuring an identical, constant nucleotide fraction. We explore DNA lengths from 10 to 80 NTs, and we design and permute sequences to tune the degree of intra- and inter-strand hybridization, from ss to hairpins to full ds (see Fig. 1A). The complete set of DNA oligomers employed in this study is reported in Supplementary Table S2. We evaluate their behavior in solution with the pL22 peptide from *Thermus thermophilus*’ ribosome (see Fig. 1B and Supplementary Table S3), a fragment buried in the large ribosomal subunit, which naturally interacts with RNA and whose sequence is highly conserved across the *bacteria* and *archaea* domains [5]. The choice of this peptide is motivated by its average composition, with polar, apolar, and hydrophobic amino acids, and a moderate fraction of charged residues. Indeed, its low *Sequence Charge Decoration* (SCD) value [67], resulting from interspersed positively charged LYS and ARG residues and from the presence of a negatively charged ASP, makes it a more realistic representative of DNA-binding domains (see Supplementary Fig. S1) than the typically used polylysine, and thus better suited to study the effects of DNA structure on coacervation.

### Characterization of the phase behavior

The stability and properties of NA/protein condensates are known to be affected by the polyelectrolyte charge ratio [41, 50, 68]. Here, to compare the behavior of NAs of different lengths and structures, we consider electro-neutral mixtures. In all the experiments, we use a peptide concentration $C^{exp}_{pL22}=300\, \mu$M.

**Figure 2.**
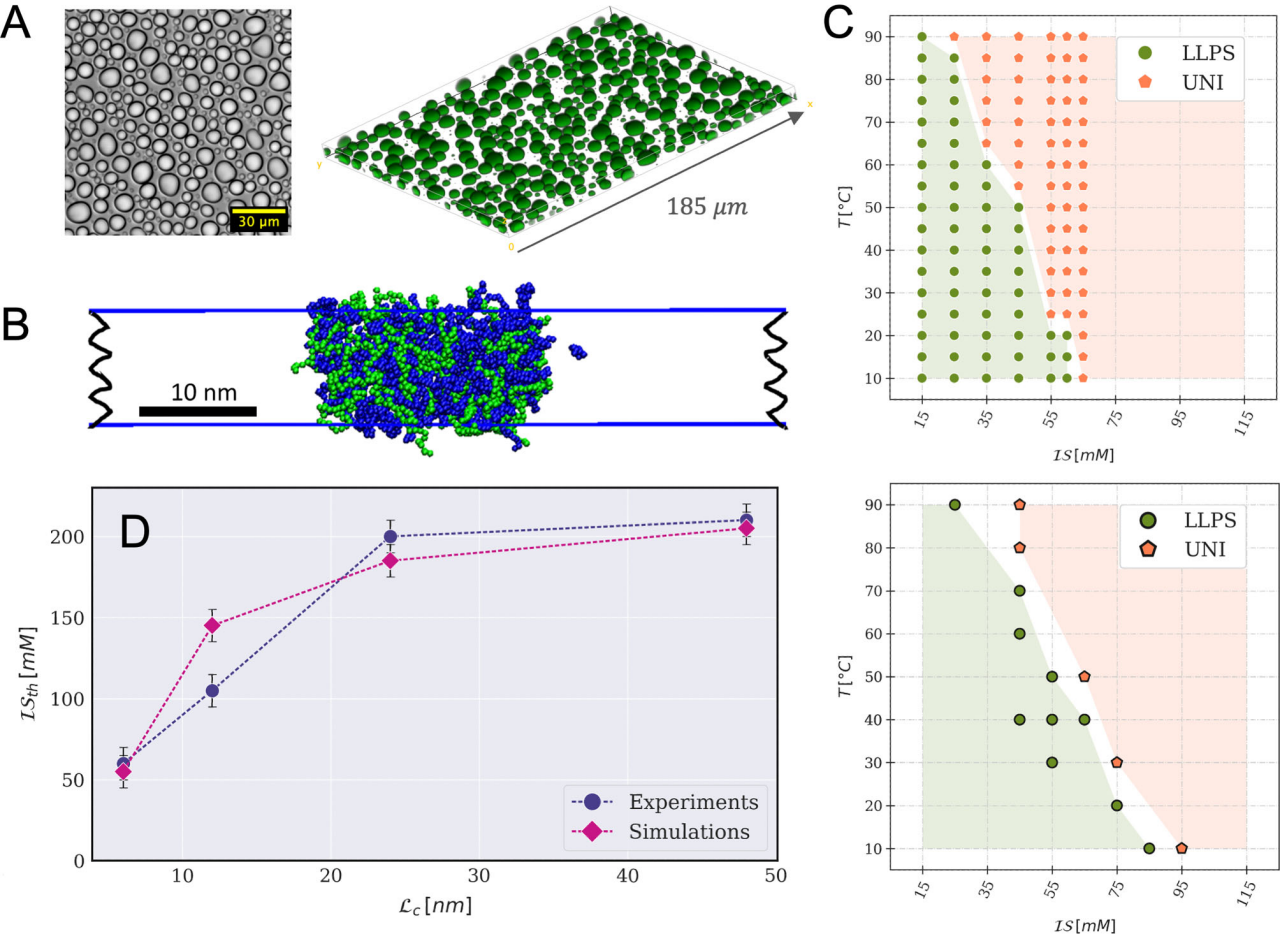
Characterization of the phase behavior of peptide/ssDNA mixtures. (**A**) Widefield microscopy image of pL22/ssDNA-20 coacervates (at $\mathcal {IS}=15$ mM and $T=298$ K) sedimented on the substrate (left); confocal 3D-slab reconstruction (right) of the same sample with 1:200 FITC-labeled DNA strands. (**B**) Snapshot from a CG-MD slab simulation of pL22/ssDNA-20 (blue and green, respectively) at $\mathcal {IS}=75$ mM and $T=303$ K. (**C**) Experimental (top) and simulated (bottom) phase diagrams of pL22/ssDNA-10, as a function of the ionic strength $\mathcal {IS}$ and of the temperature $T$; stoichiometric concentrations are: $C^{exp}_{ssDNA-10}=220\, \mu {\rm M}$, $C^{sim}_{ssDNA-10}=80$ mM, $C^{exp}_{pL22}=300\, \mu {\rm M}$, $C^{sim}_{pL22}=120$ mM. Green full circles (labeled "LLPS") and orange pentagons (labeled "UNI") denote bi- and mono-phasic states, respectively, observed in the sampled conditions. Green and orange shades indicate the extent of the two regions. (**D**) Highest ionic strength at the boundary of the LLPS region for $T=298$ K observed in experiments (blue) and simulations (violet), as a function of the contour length $\mathcal {L}_c$ of ssDNA strands with 10, 20, 40, and 80 NTs.

While both DNA oligomers and peptides are fully soluble in the buffer as single species, their mixtures spontaneously separate, already at a total concentration of $\sim 500\, \mu$M, into concentrated, liquid-like droplets, enriched in both DNA and peptides, in coexistence with a dilute supernatant (see Fig. 2A). The phase boundaries and the concentration of DNA and peptide within the phases depend on the temperature $T$ and on the total ionic strength $\mathcal {IS}$ of the mixtures. A typical bell-shaped phase boundary in the concentration-ionic strength plane is observed [3] (Supplementary Fig. S2). For a given concentration, both increasing temperature and ionic strength destabilize the condensed phase, as shown in Fig. 2C for a ssDNA 10mer. Henceforth, to characterize the coacervation propensity of different ONTs, we will compare the highest ionic strength at which the biphasic region is observed for each system at room temperature, $\mathcal {IS}_{th}$. Although this value does not correspond to the true critical point located at the maximum of the spinodal line, it is a good proxy for it, because the topology of the phase boundaries remains unaffected [24].

LLPS is also observed in the CG simulations. Fig. 2B reports the phase diagram calculated for the ssDNA 10mer, which is in remarkable agreement with the experimental phase diagram: the biphasic region appears only for $\mathcal {IS}$ values below 100 mM and narrows with increasing temperature. Compared with previous studies of DNA oligomers with highly charged polycations, such as polylysine and polyarginine, in which LLPS up to $\mathcal {IS}\sim 800-900$ mM was reported [30, 31], the system exhibits a salt resistance much closer to physiological levels, a behavior that can be attributed primarily to the more distributed charged units in pL22, similar to natural proteins.

### Effect of DNA length and secondary structure

**Figure 3.**
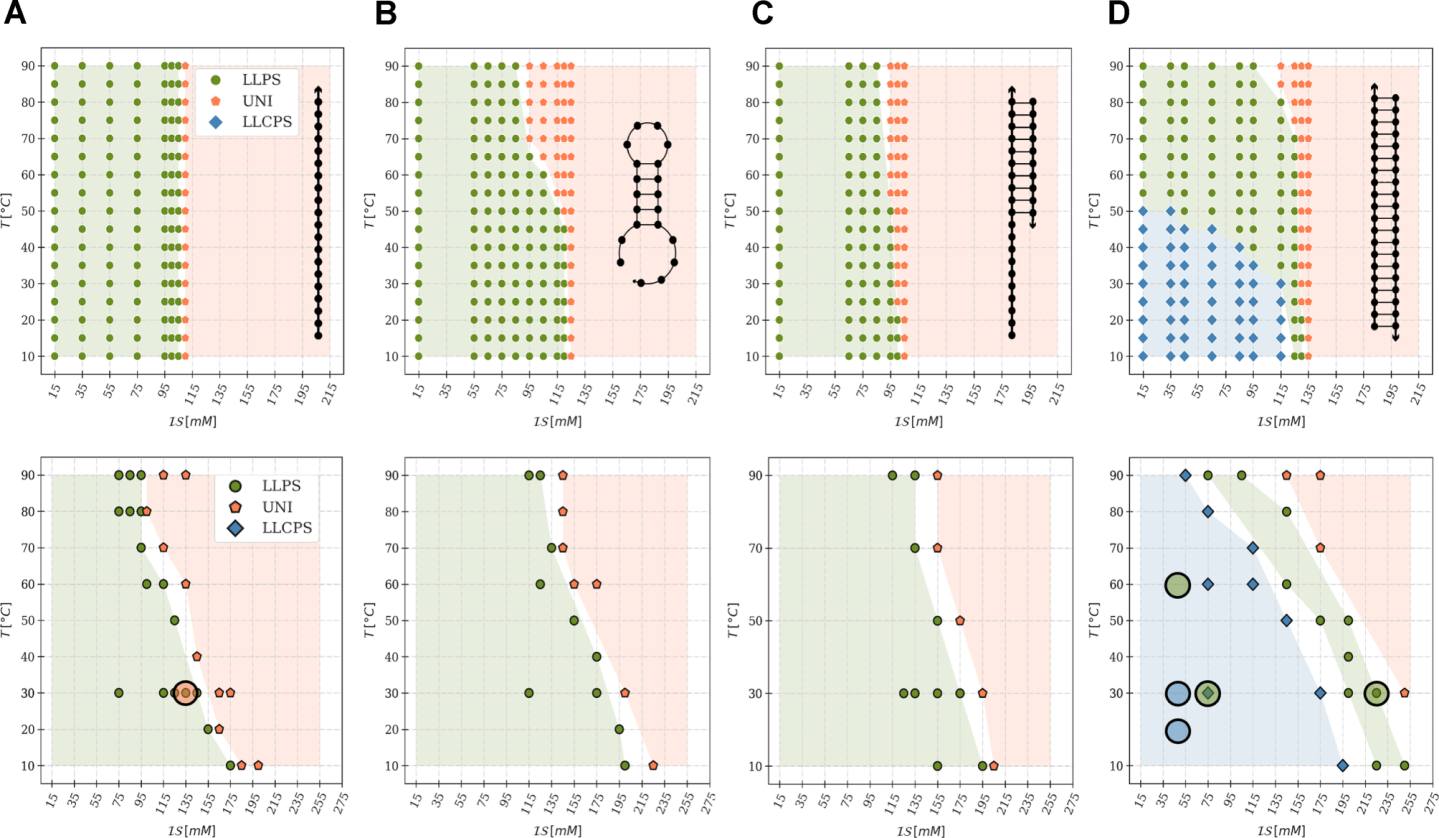
Experimental (top) and simulated (bottom) phase diagrams of mixtures of pL22 with oligonucleotides with different degrees of hybridization, as sketched in each panel. (**A**) ssDNA-20, $C^{exp}_{DNA}=110\, \mu$M & $C^{sim}_{DNA}=40$ mM, (**B**) hp5DNA, $C^{exp}_{DNA}=110\, \mu$M & $C^{sim}_{DNA}=40$ mM, (**C**) hdsDNA, $C^{exp}_{DNA}=75\, \mu$M & $C^{sim}_{DNA}=28$ mM, (**D**) dsDNA-20, $C^{exp}_{DNA}=55\, \mu$M & $C^{sim}_{DNA}=20$ mM. In all cases, $C^{exp}_{pL22}=300\, \mu {\rm M}$, $C^{sim}_{pL22}=120$ mM, so as to achieve overall charge neutrality of the mixtures. The phase behavior is denoted by the color and shape of markers and shaded regions: monophasic (orange pentagons, labeled “UNI”), isotropic biphasic (green full circles, labeled “LLPS”), and liquid crystalline biphasic (blue diamonds, labeled “LLCPS”). The black circles in the simulation diagrams in panels A and D mark states sampled using a modified bending constant for the oligomer model (2 and 4 kcal/mol$\cdot$rad$^2$ for ssDNA-20 and dsDNA-20, respectively, see Eq. S6); the color filling denotes the new observed phase-state.

The analysis of systems that differ only in the length of ssDNA allows us to dissect the contribution of NA chain connectivity in condensate formation independently of the interactions between nucleotides and amino acids. In Fig. 2D, we plot $\mathcal {IS}_{th}$ as a function of the contour length ${\mathcal {L}}_c$ of ssDNA filaments (the experimental and simulated phase diagrams of ssDNA-20 are shown in Fig. 3, while the other related diagrams are reported in Supplementary Data, Supplementary Fig. S3). In both experiments and simulations, we first observe an increase of $\mathcal {IS}_{th}$ for short ONTs, which then levels off beyond ssDNA-40, in agreement with previous reports on different NA/peptide systems [41, 42, 50, 69, 70]. We rule out sequence effect by verifying that a scrambled version of ssDNA-20, retaining an unpaired conformation, has very similar behavior (data not shown).

For both neutral polymers and mixtures of oppositely charged polyelectrolytes, an increase in chain length favors phase separation. Such growth and saturation, which reflect the decrease in the translational entropy contribution to the mixing free energy, are also predicted by classical mean-field theories of phase separation [71], although these miss important aspects of polyelectrolyte physics [38, 72].

Next, to investigate the role of DNA secondary structures in LLPS, we progressively introduce ds portions into the filaments, without affecting the base composition. Fig. 3 shows the measured and simulated phase diagrams for mixtures of pL22 with ssDNA-20 (A); with hp5DNA, a 5 bp-stem hairpin obtained by reshuffling the sequence of ssDNA-20 (B); with hdsDNA, a half-structured ONT, i.e. a ds 10mer with an unpaired tail of 10 NTs (C); and with fully base-paired dsDNA-20 (D). We observe LLPS in all of our samples, with an overall increase in stability from ssDNA-20 to dsDNA-20. This confirms the important role of the secondary structure, which, however, was never quantitatively addressed.

Compared to single filaments, base-paired structures are stiffer, with a much higher persistence length ($\sim 50$ nm for dsDNA versus $\sim 0.75$ nm for ssDNA at 10 mM Na$^+$) [73]. They also have fewer exposed aromatic nucleobases, which can engage in short-range interactions with the peptide. Both features would be expected to destabilize the coacervates for ds structures [37–39]. On the other hand, previous experiments on polylysine mixed with partially or fully hybridized DNA structures (with significant variations in base composition) [30, 31] found a sharp transition from liquid coacervates to solid precipitates whenever the ds portion exceeded 40%. This behavior was ascribed to the higher charge density of ds structures, interacting with the highly charged polylysine. Partially hybrid structures, such as hp5DNA, could combine the advantages of both high charge density and relatively high flexibility, as was suggested by the observation of particularly high resistance to their release from complexes with polylysine [74].

**Figure 4.**
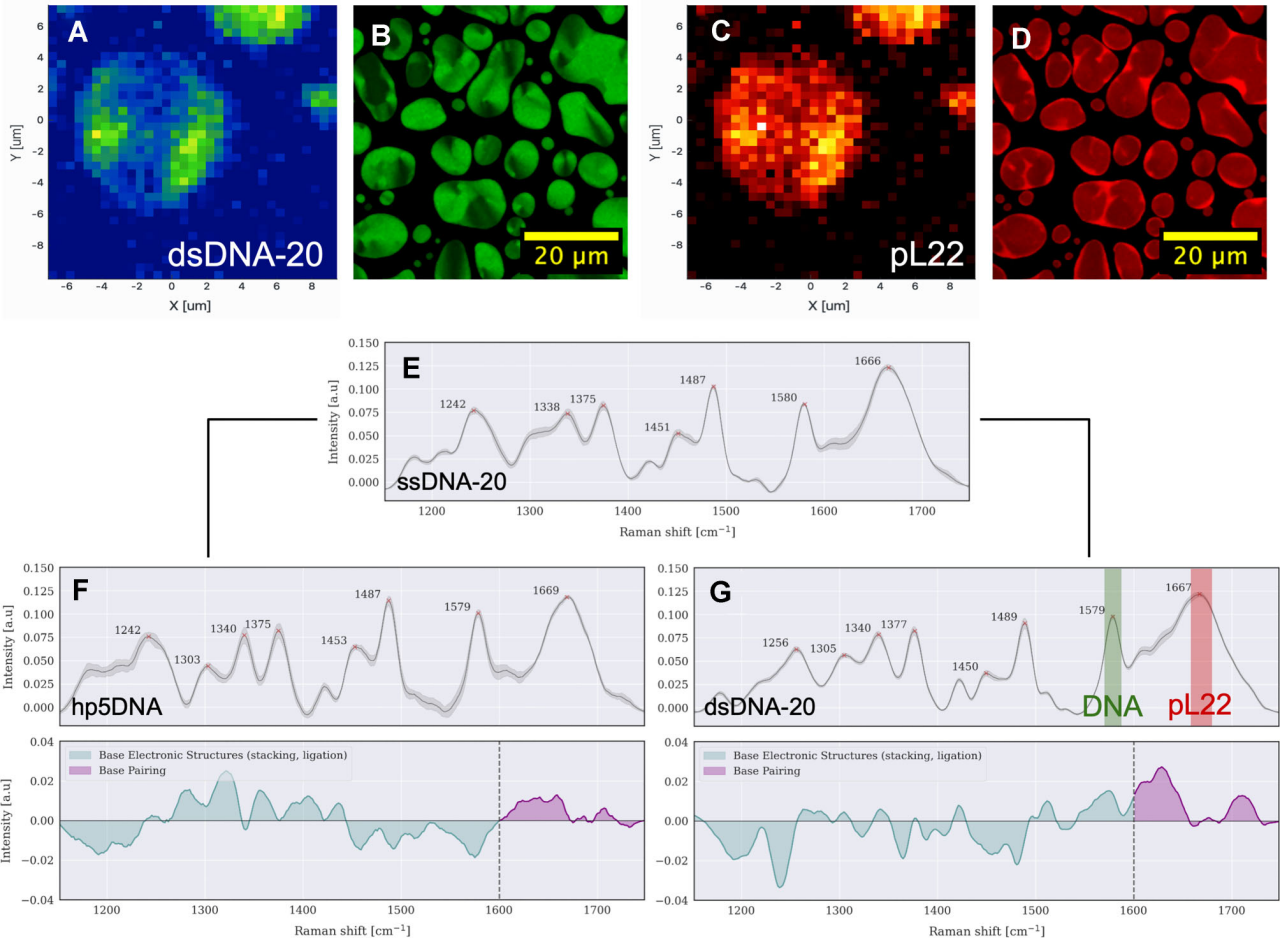
Experimental characterization of molecular arrangement and secondary structure inside the condensates. (**A–C**) Raman microscopy images of a coacervate droplet of pL22/dsDNA-20 ($C_{DNA}=55\, \mu$ M, $C_{pL22}=300\, \mu$M, $\mathcal {IS}=15$ mM, $T=298$ K). The color scales correspond to the integrated intensity of the two bands highlighted in the spectrum of dsDNA (panel G), around 1580 cm$^{-1}$ for DNA ring stretching (green) and around 1660 cm$^{-1}$ for peptide Amide I (red). (**B** and **D**) Confocal images of pL22/dsDNA-20 coacervates ($C_{DNA}=55\, \mu$M, $C_{pL22}=300\, \mu$M, $\mathcal {IS}=75$ mM, $T=298$ K). SYBR Green intercalating dye marks the anisotropic arrangement of DNA strands (B), while a fraction of peptides is labeled with TAMRA (D). (**E**) Average Raman spectra and variations inside the droplets of pL22 ($C_{pL22}=300\, \mu$M) with ssDNA-20 ($C_{DNA}=110\, \mu$M). (**F** and **G**) Average Raman spectra and variations inside the droplets of pL22 ($C_{pL22}=300\, \mu$M) with hp5DNA ($C_{DNA}=110\, \mu$M) (F) and dsDNA-20 ($C_{DNA}=55\, \mu$M) (G) at $\mathcal {IS}=15$ mM and $T=298$ K. The differences between pairs of spectra (last row) indicate in both cases that DNA strands in the coacervates are hybridized.

In addition to its higher $\mathcal {IS}_{th}$ for LLPS, dsDNA-20 differs from less structured DNA strands in the appearance of a liquid crystalline phase within the demixed droplets (light blue region in Fig. 3D) [31, 32, 35]. This ordered yet fluid phase transitions to the isotropic liquid state at temperatures above $\sim 50^\circ$C or close to the ionic strength threshold. ONTs and peptides are colocalized in droplets, as assessed by Raman microscopy (Fig. 4A and C): the intensities of DNA- and peptide-specific bands in the Raman spectrum [75, 76] (Fig. 4G) display a correlated, non-uniform spatial distribution of the two biomolecules. Moreover, the anisotropic arrangement of DNA helices, a hallmark of the LC phase, is evidenced by droplet birefringence (Supplementary Fig. S4) and by the anisotropic fluorescent emission of an intercalated dye, SYBR green. In Fig. 4B, the confocal microscopy image displays alternating bright and dark regions within the fan-shaped domains (suggesting a columnar phase) because the horizontally polarized incident laser has maximum (minimum) efficiency of excitation for horizontal (vertical) dye molecules, corresponding to vertically (horizontally) lying dsDNA helices. In contrast, no fluorescence anisotropy is observed for dye-labeled peptides (Fig. 4D), a sign of their overall disordered arrangement.

The experimentally observed trends of the mixtures of pL22 with ONTs of different secondary structures align with those in the simulated phase diagrams (bottom row in Fig. 3). In particular, dsDNA-20 exhibits the same sequence of LC and isotropic liquid regions at increasing $\mathcal {IS}$ and $T$. The calculated radial distribution functions (Supplementary Fig. S5), which are distinctly different from those computed in the presence of polylysine, demonstrate the (isotropic or anisotropic) liquid nature of the concentrated phase.

Despite the general agreement between experimental and simulated phase diagrams, one can notice that the stability of the LLPS region is overestimated in the latter, in particular for the LC region of dsDNA. One source of this discrepancy is the different stoichiometric concentration used in experiments and simulations (Supplementary Fig. S2). At low ionic strength, both fall within the biphasic region, but the highest ionic strength at which such a region is observed may be different for the two concentrations [24]. In fact, lower ${\mathcal {I}S}_\mathrm{th}$ was predicted by test simulations performed at the experimental concentration (Supplementary Fig. S6). We must also mention the limitations of our CG model. In particular, the same parameterization for NT–AA interactions is used for paired and non-paired nucleotides; as a consequence, reduced exposure of the bases in structured DNA is ignored [77], which may lead to an overestimate of hydrophobic interactions, particularly relevant for dsDNA and at higher ionic strengths.

Discrepancy between the experimental and simulated phase boundaries could also derive from partial unfolding of hybridized ONTs in the experimental samples [44]. Indeed, while hp5DNA is stable up to around $50\, ^{\circ }$C, hdsDNA melts around $40\, ^{\circ }$C (Supplementary Fig. S7). However, Raman spectra highlight the presence of base pairing and stacking signatures within the coacervates at room temperature [75, 76] (Fig. 4 and Supplementary Fig. S8): the average intensities in the 1150–1750 cm$^{-1}$ interval show significant differences for both hairpins and double helices with respect to the corresponding signals from ssDNA (last row in panels F and G, respectively). Raman spectra therefore confirm that the ONTs mostly retain their hybridized form inside the condensates, establishing a link between DNA structure and phase stability.

### Quantifying charge distribution and flexibility of NA oligomers

In order to describe the observed trends in the phase behavior of various ONTs and connect them to single-chain properties, we must properly address the molecular features of ONTs: while length is a well-known determinant of phase behavior of DNA and polyelectrolytes in general [70], flexibility and charge density have opposite effects, but the quantities typically used to measure them can defy intuition, are often interconnected, or fail to capture subtle yet important differences. We briefly introduce here our approach to define simple but meaningful parameters to describe the critical NA properties that determine their coacervation.

#### Length

Because we compare the behavior of ss and ds ONTs, or their combination, the number of nucleotides is not an ideal metric. To consider the different building blocks, we rather consider the contour length ${\mathcal {L}}_c$ of the various DNA strands, defined as the sum of ss and ds steps, of length 0.6 nm and 0.34 nm, respectively [78].

#### Flexibility

Stiffness is easily defined in long, semi-flexible polymers in terms of persistence length, i.e. the typical correlation length of the direction of polymer axis, but in oligonucleotides, the very definition of persistence length becomes meaningless, even more for partially folded structures. The related concept of Kuhn length, which represents the length of each segment of a freely-jointed chain, also fails to describe short, heterogeneous strands, and results depend on the ONT length [64]. Indeed, the definition of a parameter to characterize the local flexibility of real polymeric systems with different architectures is far from obvious. A simple bending rigidity parameter cannot take into account secondary structure and sample conformational fluctuations in an effective way. We find that a suitable quantity able to capture the overall flexibility is the Root Mean Square Deviation (RMSD) of the atomic positions from their average value, normalized by the number of monomers to decouple the residual linear length dependence (Eq. 1 and Supplementary Fig. S10). In practice, for NAs, we use the coarse-grained representation provided by *oxDNA* (see Supplementary Data and Supplementary Fig. S9).

#### Charge density

It is quite natural to think that dsDNA has a higher charge density than ssDNA because the two strands are paired, which in turn strongly affects their arrangement and stiffness. However, a quantitative and applicable definition of charge density requires determining an appropriate length, surface, or occupied volume, which is challenging for heterogeneous structures with spatially distributed charges. We find that the absolute value of the average electrostatic potential $|\Phi _e|$, experienced by a probe charge moving along the surface of an atomistic representation of the ONT, being sensitive to the spatial distribution of charges, can capture such effects. To take into account the intrinsic dependence of the average electrostatic potential on the polymer length (see Supplementary Data), we use the normalized quantity $|\widetilde{\Phi }_e|$ (Eq. 2).

**Figure 5.**
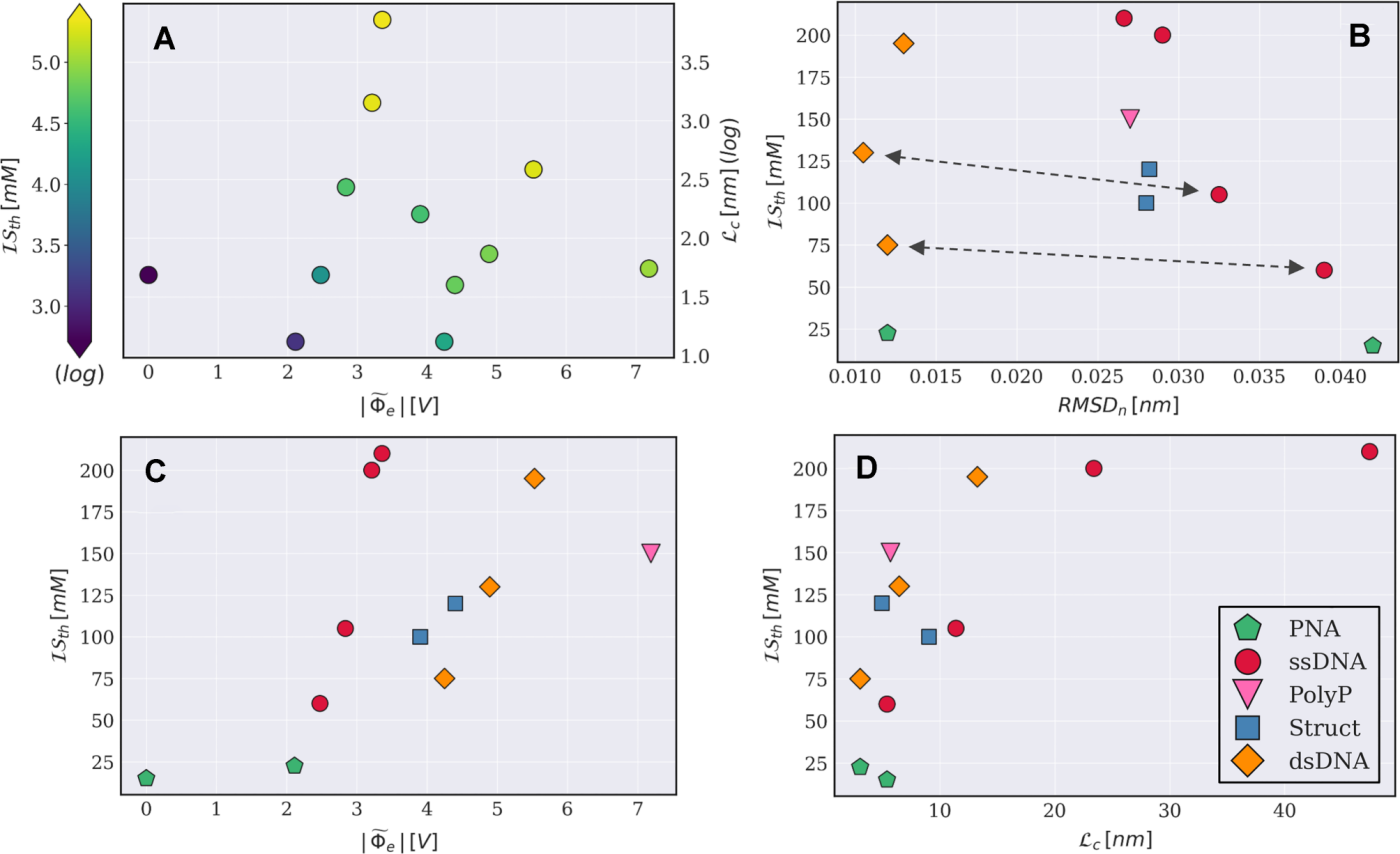
Experimental ionic strength threshold $\mathcal {I}S_{th}$ at the LLPS boundary at 298 K, for mixtures of pL22 with ONTs, PNA, and polyP, as a function of the contour length ${\mathcal {L}}_c$, of the normalized average electrostatic potential $|\widetilde{\Phi }_e|$ and of the normalized $RMSD_n$ of the polyanions. (**A**) Projection on the $\left(|\widetilde{\Phi }_e|,{\mathcal {L}}_c\right)$ space with color-coding of the ionic strength (contour length and color bar in log scale). (**B-D**) Dependence of $\mathcal {I}S_{th}$ on: (**B**) $RMSD_n$, (**C**) $|\widetilde{\Phi }_e|$ and (D) $\mathcal {L}_c$. Colors and markers correspond to different families of structures, as indicated in the caption (panel D, bottom right). Arrows in panel B highlight the pairs ssDNA-10/dsDNA-10 and ssDNA-20/dsDNA-20 (lower and upper arrow), characterized by lower/higher values of $|\widetilde{\Phi }_e|$ and higher/lower $RMSD_n$.

### Charge distribution determines the salt resistance of condensates

In Fig. 5, we plot the experimental $\mathcal {IS}_{th}$ as a function of the three parameters defined above for different ONTs, grouped according to their secondary structure.

The strong dependence of condensate stability on NA length, shown in Fig. 2D for ss ONTs, is also exhibited by ds ONTs (Fig. 5D), but the latter, which are less flexible and more charged, display systematically higher $\mathcal {IS}_{th}$ values than ss of similar contour length. In the literature, theories on long polyelectrolytes [29, 38, 39] and experimental reports on ONTs with varying sequence compositions [31] agree on a stabilizing effect of chain flexibility on LLPS. However, the same does not hold when comparing ss and ds ONTs, as shown by the arrows in Fig. 5B: namely, the salt resistance of ssDNA-10/20 is slightly *smaller* than that of dsDNA-10/20 despite the much *larger *$RMSD_n$. Clearly, other factors come into play here. Indeed, Fig. 5C shows that larger $|\widetilde{\Phi }_e|$ values positively correlate with enhanced LLPS – with partially hybridized structures in between ss and ds – quantitatively establishing the major role of charge distribution.

The trade-off between flexibility and charge density can also be appreciated in the partially hybridized structures hp5DNA and hdsDNA, which show salt resistance similar to ssDNA-20 and intermediate between dsDNA-10 and dsDNA-20. In particular, the hairpin structure, besides being more stable than unstructured single strands, combines highly flexible tracts with large charge density and thus maximizes LLPS propensity. The overall trend is well captured by the parametric plot in the $\left(|\widetilde{\Phi }_e|,{\mathcal {L}}_c\right)$ space (Fig. 5A), with increasing stability for longer and/or more charged NAs.

In simulations, it is possible to tune flexibility without affecting charge distribution, to isolate its role. Flexibility can be effectively modulated through a bending potential between adjacent beads of a chain, independently of the other interactions, of temperature, and of ionic strength (see Eq. S6). We observe that stiffening ssDNA-20 (by adding a bending constant of 2 kcal/mol$\cdot$rad$^2$) reduces the width of the LLPS region (orange-filled circle in Fig. 3A). This is in agreement with theory [37, 38] and simulations [39] based on generic models. Conversely, loosening dsDNA-20 (by reducing the bending constant from 40 kcal/mol$\cdot$rad$^2$ to 4 kcal/mol$\cdot$rad$^2$) does not significantly affect the LLPS boundary but strongly reduces the propensity to LC ordering (blue- and green-filled circles in Fig. 3D).

### Extension to NA analogs and higher-order structures

**Figure 6.**
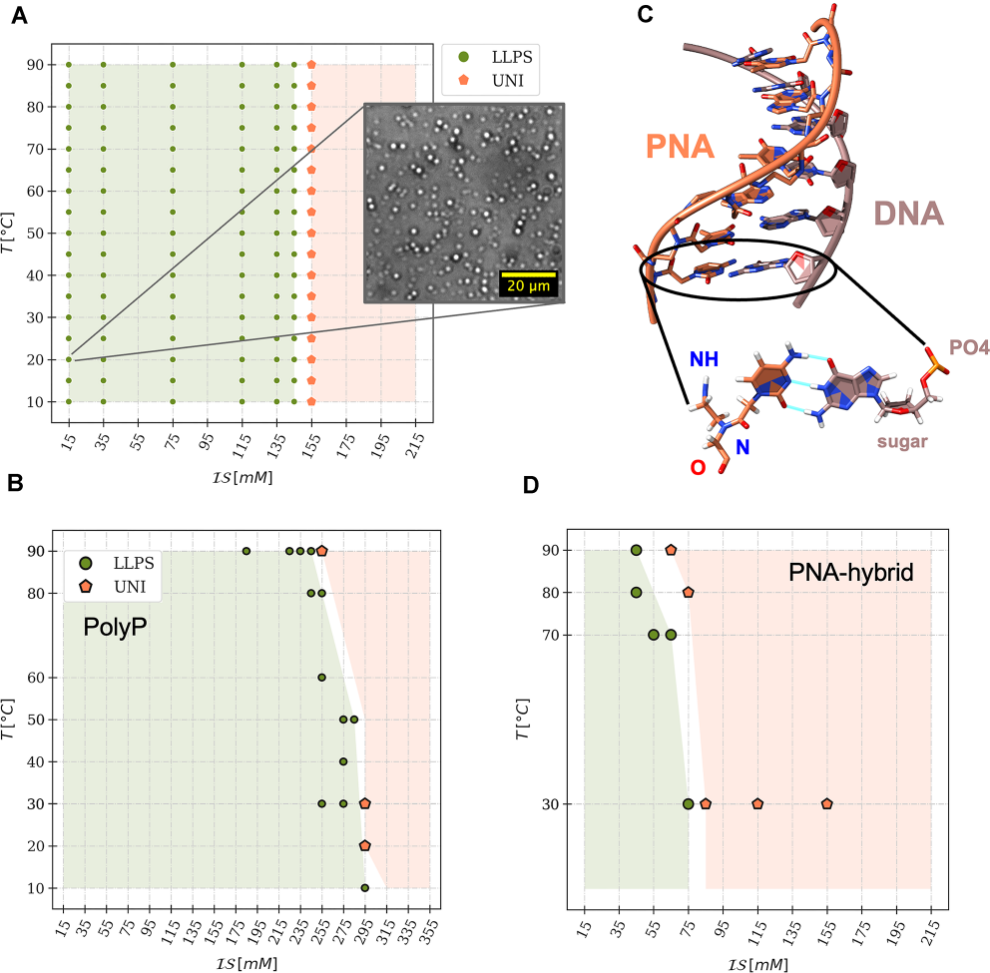
Coacervation of NA analogs. (**A** and **B**) Experimental (**A**) and simulated (**B**) phase diagrams of pL22/polyP mixtures at stoichiometric concentrations $C^{exp}_{polyP}=110\, \mu$M, $C^{sim}_{polyP}=40$ mM, $C^{exp}_{pL22}=300\, \mu$M, $C^{sim}_{pL22}=120$ mM. The phase behavior is denoted by the color and shape of markers and shaded regions: monophasic (orange pentagons), isotropic biphasic (green full circles), and liquid crystalline biphasic (blue diamonds). The inset in A shows a widefield micrograph of the system at low ionic strength and ambient temperature. (**C**) Structure of a PNA-10 hybrid (PDB 1PDT); as the C-G WC pairing shows, PNA and DNA share canonical nucleobases, but the deoxyribose-phosphate backbone of DNA is replaced by a pseudopeptide skeleton in PNA. (**D**) Simulated phase diagram for mixtures of pL22 ($C_{pL22}=120$ mM) with the PNA-20 hybrid ($C_{PNA-20}=40$ mM).

The overall trend summarized in Fig. 5 suggests that LLPS is enhanced for longer strands and, for a given contour length, electrostatic interactions prevail over flexibility. To further test the generality of this description, we extend our investigation to NA variants, separating the contribution of the charged backbone from the base pairing moieties and better decoupling flexibility from charge density. To this end, we mix pL22 with (i) polyphosphates, as a model for the NA backbone [55, 56], (ii) PNA filaments, which combine an uncharged peptidic backbone with nitrogen bases, and (iii) hybrid ds helices formed by a DNA strand and a complementary PNA filament [52], with flexibility similar to dsDNA but half its charge content (Fig. 6C).

While the neutral PNA does not display LLPS under any condition, a polyP 20mer forms liquid droplets following the general trend found for DNA oligomers (Fig. 5), but with strikingly different properties. Indeed, polyP features both high charge density and high flexibility and was previously shown to phase separate in mixtures with other positively charged peptides and proteins [56, 79–81]. Interestingly, we find that it displays higher salt resistance than dsDNA-20, but with markedly smaller droplets both at low and high ionic strength (Fig. 6A). Simulations based on a CG model of polyP, built in analogy with the model used for DNA (see Materials and Methods), also predict high stability and confirm the major role played by the charge density (Fig. 6B). However, as previously observed in dsDNA, the threshold ionic strength is overestimated compared to experiments; more tailored parameterization may be required to achieve quantitative agreement.

As regards DNA-PNA hybrids, experimental investigation is only possible for 10mers, because of the solubility limits of longer strands. For such a molecule, no LLPS is observed at the same concentrations and solvent conditions of ssDNA-10 and dsDNA-10 (Supplementary Data, Supplementary Fig. S12). However, simulations are extended to a 20mer to directly compare it to its ss and ds DNA counterparts (Fig. 6D). For the PNA strand, the same CG model of ssDNA is used, with charges set equal to zero. The DNA-PNA hybrid exhibits lower $\mathcal {IS}_{th}$ than ssDNA, which has a very similar charge density but is much more flexible. As expected for its reduced electrostatic potential, it is also much less stable than dsDNA. Moreover, LC ordering is suppressed for pL22/PNA-20 hybrid.

Based on the experimental and simulated trends for DNA and single NA components (Figs. 5A and 6), it would be possible to quantitatively predict the boundaries of LLPS behavior for other NAs, canonical and non-canonical forms, for which we can estimate $\mathcal {L}_c$, $RMSD_n$ and $|\widetilde{\Phi }_e|$. For example, folded structures such as G-quadruplexes, which are much stiffer and have a larger electrostatic potential than dsDNA (Supplementary Fig. S11), have been shown to display higher condensate stability, while easily giving rise to solid aggregates [40].

### The onset of liquid crystalline ordering

dsDNA endows coacervates with a distinct sensitivity to the state variables, which is tuned by the polycations. In mixtures of dsDNA oligomers with highly charged amino-based polycations or peptides, liquid coacervates at high NaCl concentration ($\gtrsim 500$ mM) transform into precipitates at low ionic strength [30]. In mixtures with polylysine [31, 32] a richer phase diagram was reported, including various LC phases, again with the isotropic liquid phase only present at high salt concentration ($\gtrsim 800$ mM) or high temperature. Here, with pL22, we detect LC ordering in a wide range of temperatures and nearly physiological ionic strengths (see Figs 3D and 4B and D); unlike the previous studies, at room temperature, the isotropic phase appears already at a relatively low ionic strength, $\mathcal {IS}\sim 100$ mM.

Theories and simulations based on generic models of oppositely charged polyelectrolytes [62, 82, 83] predict the occurrence of a first-order isotropic-to-LC transition inside coacervate droplets, driven by short-range anisotropic (excluded volume) interactions and by electrostatic interactions, if at least one of the polyelectrolytes has weak flexibility and a sufficiently high aspect ratio. Previous slab simulations of dsDNA-containing coacervates [41] missed the onset of LC ordering, probably because of a different modeling of oligonucleotides; in fact, stiffness and aspect ratio of the latter turn out to be crucial to this purpose.

**Figure 7.**
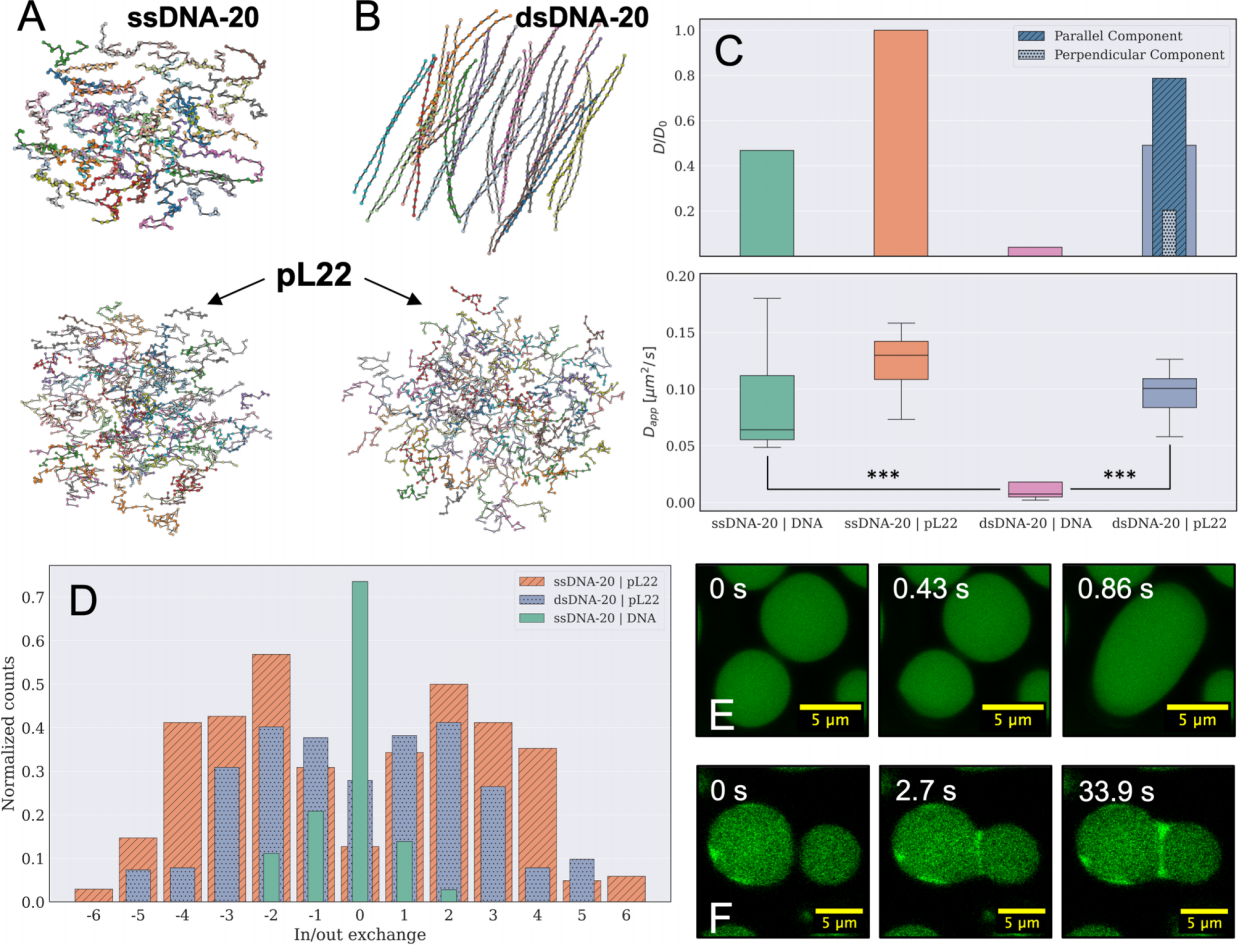
Ordering and dynamics of DNA and peptides inside isotropic and liquid crystalline condensates. (**A** and **B**) Configurations of DNA oligomers (top) and peptides (bottom) from MD trajectories of mixtures of pL22 with ssDNA-20 (**A**) and dsDNA-20 (**B**). Simulations are performed at $\mathcal {IS}=75$ mM and $T=303$ K, matching the experimental conditions. (**C**) Diffusion coefficients of pL22 and DNA oligomers extracted from simulations (top) and from FRAP measurements (bottom) on isotropic condensates of ssDNA and LC condensates of dsDNA; simulation data in the barplot are rescaled by $D_0$, the diffusion coefficient of pL22 in isotropic condensates. For pL22 in LC condensates, the contributions from the perpendicular and parallel directions of motion with respect to the director are shown and marked with different hatches and colors. The experimental boxplot results from the fitting of $10 \le n \le 14$ independent recovery curves from different droplets for each of the two samples (ssDNA-20 & dsDNA-20) and two channels (pL22-TAMRA & DNA-FAM). The *** denotes a *P*-value less than 0.001 in a Mann–Whitney U two-sided test. (**D**) Distribution of in (positive)/out (negative) exchanges from molecular clusters, calculated for pL22/ssDNA-20 and pL22/dsDNA-20 condensates during the last 3 $\mu$s of MD trajectories. Counts of exchange events are normalized by the number of molecules in the box for each species. No exchange is observed for dsDNA-20 in the pL22/dsDNA-20 LC phase (not shown). (**E** and **F**) Confocal images of fusion events for droplets of pL22 and fluorescently labeled ssDNA-20 (**E**) and dsDNA-20 (**F**).

Remarkably, we find the LC phase in our CG simulations (Fig. 3D). Fig. 7A and B show two configurations, extracted from MD trajectories, of peptides and NAs in isotropic condensates formed by ssDNA-20 and in LC condensates formed by dsDNA-20. While rod-like ds ONTs are on average aligned along a common direction within the LC phase, coiled ss ONTs are randomly oriented in space. Surprisingly, such disparity does not appear for the peptides, for which we cannot detect a significant degree of order in either case.

Further quantitative insight into the molecular organization inside the coacervates is provided by the radial distribution function (rdf) for the amino acids and the NTs in different mixtures, quantifying the degree of spatial correlation of the different species (see Supplementary Fig. S5). In isotropic coacervates (ssDNA and dsDNA at high $\mathcal {IS}$), rdfs indicate direct contacts between amino acids and nucleotides, but no contacts between the nucleotides and a few contacts between peptides. Instead, in LC coacervates of dsDNA at low $\mathcal {IS}$, the rdfs for DNA-DNA and peptide-DNA pairs provide clear evidence of two coordination shells. In a control case of dsDNA mixed with a polylysine 18-mer, a pronounced, long-range order is observed (Supplementary Fig. S5), which, together with the very low mobility of the peptide chains, aligns with the experimental observation of solid precipitates in these systems [30, 39].

Taking advantage of the molecular-scale information provided by the MD simulations, we can discuss the various factors contributing to the onset and stabilization of LC order inside dsDNA coacervates. The fact that NA flexibility tunes the LC stability (Fig. 3D) is consistent with previous predictions of theory and simulations [62, 83]. Also the decrease in density at the LC-isotropic transition aligns with these studies, suggesting an entropy-driven mechanism for LC formation, associated with a decrease in excluded volume of the rod-like units upon ordering [84], as often observed for lyotropic LCs. However, the detrimental effect of salt on the stability of the LC phase suggests a role of electrostatic interactions. Indeed, in simulations of PNA-DNA hybrid, which has a very similar shape anisotropy to the dsDNA oligomer, LC ordering is suppressed despite the much higher local concentration. In MD simulations of salt-free coacervates of semiflexible polyelectrolytes, with a generic CG model lacking any molecular details [62], electrostatic interactions, tuned through the charge of the polycations, were found to facilitate the emergence of LC ordering. According to theoretical studies of salt-free mixtures [82, 83], fluctuation-induced attractive Coulomb correlations between the rodlike polyanions would be responsible for the enhancement of a weakly ordered nematic phase in low-density coacervates.

To ascertain the role of peptides in the emergence of LC ordering in our systems, we estimate the average number of contacts, $N_c$, of the amino acids of pL22 with nucleotides inside isotropic and LC coacervates (see Supplementary Fig. S13). Overall, the pattern of contacts mirrors the charge of amino acids, as reported for proteins [28] and mixtures of peptides with relatively short DNA duplexes [85]. However, for dsDNA $N_c$ is higher in the LC (Supplementary Fig. S13B) than in the isotropic phase (Supplementary Fig. S13C), which in turn has a $N_c$ profile close to the isotropic ssDNA (Supplementary Fig. S13A). This is consistent with the fact that the two phases, with the same NT/AA ratio (although the ratio of peptides to NA chains in the former is twice as big as in the latter), also display a similar density (see Supplementary Fig. S5). Interestingly, in the LC phase the interrelated increase of density and onset of orientational order not only bring about an increase of peptide-DNA contacts, but also an increase in the number of DNA-DNA bridges enforced by peptides (see Supplementary Fig. S14).

In summary, our simulations suggest a crucial and subtle role of electrostatic interactions for the mesomorphic behavior of peptide/dsDNA coacervates, where peptides contribute both through direct interaction with dsDNA and by mediating DNA–DNA interactions. LC ordering thus entails a strengthening of electrostatic interactions, which in turn stabilizes the ordered phase. If the electrostatic interaction grows too much, as in the case of polylysine/dsDNA-20 (Supplementary Fig. S13D), solid precipitates are predicted, in agreement with experiments. We may mention that, within our model, an increase in the ionic strength implies both a decrease in the Debye length and a decrease in the depth of the minimum of the non-bonded pair potential (Supplementary Data, Supplementary eqs. S1 and S3). In real systems, a more complex mechanism may occur, involving ion exchanges and hydration effects.

### Molecular mobility in different phases

The asymmetry in the structural arrangement between DNA and peptides is reflected in the mobility measured in simulations, which strongly depends on the molecular structure and on the environment [86]. The DNA and peptide concentrations inside simulated droplets are similar to the experimental ones, despite the different stoichiometric concentration (Supplementary Fig. S3). However, because in CG models the diffusion time scale is accelerated due to the lack of hydrodynamic interactions and molecular details [17, 70, 87], we report scaled diffusion coefficients, taking the value for peptides in the isotropic phase as a reference. Inside isotropic droplets formed by ssDNA, or dsDNA at high $\mathcal {IS}$, the two species display a similar diffusion coefficient, with peptides slightly more mobile than DNA filaments (Fig. 7C, *top row*). On the contrary, in LC droplets of dsDNA, while the dynamics of ordered DNA duplexes are strongly reduced (see also Supplementary Fig. S15), the peptides surrounding and bridging them maintain a diffusivity similar to that in the isotropic phase, with faster motion parallel to DNA chains than perpendicular to them. This suggests that peptide bridges between DNA filaments are highly dynamic [88]. Indeed, the exchange rate between the condensates and the dilute phase (Fig. 7D) also shows a similar pattern for interface mobility, with fast kinetics of peptides for both isotropic and LC coacervates, while for dsDNA, the exchange between LC domains and the supernatant is suppressed.

To verify these simulation findings, we experimentally track the mobility of both components inside the condensates by labeling them with different fluorescent dyes and performing Fluorescent Recovery After Photobleaching (FRAP) measures [89, 90] in the isotropic and LC cases (see Supplementary Fig. S16). By locally bleaching a portion of the droplet and measuring the evolution of the fluorescent signal in the bleached region, we can separately measure the effective diffusivity of DNA and peptide (Fig. 7C, *bottom row*). Similarly to simulations, we find that while the translational diffusion of dsDNA in the LC domains is much slower than that of ssDNA in the isotropic droplets, the mobility of peptides in the two cases is very similar. Finally, the strong mobility reduction of LC dsDNA is also manifested on a larger scale, in coalescence events of coarsening droplets. As shown in confocal microscopy frames in Fig. 7E, where ONTs are tagged with a fluorescent dye, the characteristic fusion time, which depends on the viscosity (and surface tension) of the concentrated phase [50, 86], is much faster for ssDNA (top row) than for LC dsDNA (bottom row).

## Conclusion

The physical properties of nucleic acids, such as length, charge density, and flexibility, which are heavily dependent on their hybridization state, significantly influence the stability and the internal structure of biomolecular condensates. However, due to the intrinsic interdependence of such properties, it remains difficult to isolate their individual roles; indeed, conflicting interpretations of experimental behaviors exist in the literature. To address this question, we focused on well-defined model systems under controlled conditions: namely, electroneutral mixtures comprising a moderately charged, disordered peptide and a series of systematically varied oligonucleotides.

The integration of experiments with coarse-grained molecular simulations was crucial to gaining insight into key variables and underlying mechanisms that remain inaccessible to direct observation. The system-specific CG model employed here successfully captures the essential links between molecular structure and the resulting phase behavior, as well as the structural and dynamic properties of the coacervates. Nevertheless, direct comparison with experiments also reveals certain limitations that warrant further development.

By analyzing salt resistance across different NA structures, we find that it scales positively with the degree of base pairing in the ONTs, and we quantitatively assess the dominant role of charge distribution, stemming from the phosphate backbone.

Regarding the local flexibility of NAs, which has led to contrasting conclusions in the literature, our findings suggest that, as predicted by theory and simulations, its increase may be invoked to explain the stabilization induced, for example, by variations in the sequence of single-stranded filaments [31]. However, this argument does not extend to dsONTs, as their mechanical properties are distinctly different from those of their ss counterparts. Other explanations must be explored for effects on LLPS that were ascribed to changes in bending stiffness [31, 91, 92].

While stiffness alone does not explain the LLPS stability of dsONTs, it is, conversely, key for the onset of liquid crystalline order within the coacervates. Previous studies employing dsONTs and highly charged peptides [31, 32] reported liquid crystal phases at intermediate salt concentration, between solid precipitates and liquid coacervates. Here, we demonstrate that liquid crystalline coacervates also form in the presence of moderately charged peptides, and with the support of simulations, we characterize their structural and dynamical properties. We find that inside LC droplets, mobile peptides transiently bridge stiff dsDNA tracts, retaining a disordered arrangement and high mobility. These transient bridges, coupled with the entropic gain arising from reduced excluded volume of DNA duplexes, contribute to the stabilization of the ordered phase.

The insights gained from this study are expected to have an impact on burgeoning fields, most notably NA therapeutics employing oligonucleotides with varying hybridization states, such as antisense and CpG oligonucleotides, siRNAs, and aptamers. Likewise, they offer a framework for synthetic biology and the design of protocells, where chemical modifications of NAs provide a pathway for dynamic control of spatiotemporal organization through their physical properties [93].

## Supplementary Material

gkag253_Supplemental_File

## Data Availability

The data underlying this article are available upon reasonable request from the corresponding authors.
