# Supplementary material for: Decoding nucleic acid contributions to phase separation and ordering in biomolecular condensates
Source: Nucleic Acids Res. 2026 Mar 30;54(6):gkag253. doi: 10.1093/nar/gkag253 (PMC13034049; doi:10.1093/nar/gkag253)
Supplement: gkag253_Supplemental_File [file gkag253_supplemental_file.pdf]

Supplementary data for: Decoding Nucleic Acid Contributions to Phase Separation and Ordering in Biomolecular Condensates

Daniele Asnicar[1,†], Simone Codispoti[2,†], Carlo Morasso[3], Alberta Ferrarini[1,*], and Giuliano Zanchetta[2,*]

[1]Department of Chemical Sciences, University of Padova, Via Marzolo 1, 35131 Padova, Italy
[2]Department of Medical Biotechnology and Translational Medicine, Università degli Studi di Milano, via F.lli Cervi 93, 20054 Segrate (MI), Italy
[3]Laboratory of Nanomedicine, Istituti Clinici Scientifici Maugeri IRCCS, Via Maugeri 10, 27100 Pavia, Italy
[†]These authors contributed equally

Sequence design

Sequence optimization was performed with the aid of the *NUPACK Python module* [1]. Given a seed DNA sequence, random permutations were iteratively applied and only the structural ensembles matching the desired criteria were kept. The selection criteria were based on: (i) the number of base-paired nucleotides in the *Minimum Free Energy* structure; (ii) the concentration of inter-strand constructs made up of 2 sequences; (iii) the fraction of unpaired nucleotides at equilibrium. For all sequence selections, the default *dna04* model and the *stacking* ensemble were used, and a NUPACK test tube was created containing the seed sequence at the same concentration used in the experiments; moreover, the analysis is performed at $T = 23°\text{C}$ and 100 mM NaCl, mimicking the conditions of LLPS before and after temperature annealing.

Molecular Dynamics simulations

Coarse grain model We used the CG model developed in Refs. [2–4], where peptides and NAs are represented as chains of linked soft beads, each corresponding to an amino acid or a nucleotide. Here, we extended the model to describe fully and partially double-stranded structures, including hybrid PNA-DNA duplexes, as well as polyphosphate chains. The interaction potential between a pair of beads is expressed as the sum of bonded and non-bonded terms [2].

The non-bonded potential contains a short-range (U_{HPS}) term and, for NTs and charged amino acids, an electrostatic contribution (U_{DH}). The short-range potential has the form proposed by Ashbaugh and Hatch [5] to describe the effective interaction between amino acids:

$$U_{\text{HPS}}(r_{ij}) = \begin{cases} U_{\text{LJ}} + (1-\lambda_{ij})\epsilon & \text{if } r_{ij} \le 2^{(1/6)}\sigma_{ij} \\ U_{\text{LJ}}\lambda_{ij} & \text{otherwise} \end{cases} \tag{S1}$$

where r_{ij} is the distance between the beads i and j and U_{LJ} is the pure Lennard-Jones potential

$$U_{\text{LJ}}(r_{ij}) = 4\epsilon\left[\left(\frac{\sigma_{ij}}{r_{ij}}\right)^{12} - \left(\frac{\sigma_{ij}}{r_{ij}}\right)^{6}\right] \tag{S2}$$

with ϵ a quantity that determines the absolute energy scale of the short-range interactions and σ_{ij} related to the diameters of the beads. The hydropathy parameter λ_{ij} in Equation (S1) is introduced to tune the attractive force between beads according to their hydrophobicity. At $r_{ij} = 2^{(1/6)}\sigma$ the potential takes the value $U_{\mathrm{HPS}} = -\epsilon\lambda_{ij}$ and the interaction force vanishes. At shorter distances the force is repulsive and is identical for all beads; at longer distance the force is attractive for $\lambda_{ij} > 0$ (hydrophobic interaction), whereas it remains repulsive for $\lambda_{ij} \leq 0$ (hydrophilic interaction).

The electrostatic interaction is modeled by the Debye-Hückel potential:

$$U_{\mathrm{DH}}(r_{ij}) = \frac{Z_i Z_j e^2}{4\pi\epsilon_0\epsilon_r r_{ij}} e^{-\frac{r_{ij}}{\lambda_{\mathrm{D}}}} \tag{S3}$$

where Z_i and Z_j are the charge numbers of the beads, ϵ_0 is the vacuum permittivity, ϵ_r is the (relative) dielectric constant of water, e is the elementary charge, and λ_D is the Debye screening length:

$$\lambda_{\mathrm{D}} = \sqrt{\frac{\epsilon_0\epsilon_{\mathrm{r}} k_{\mathrm{B}} T}{2000 N_{\mathrm{Av}} e^2 \mathcal{IS}}} \tag{S4}$$

with N_{Av} the Avogadro number, k_{B} is the Boltzmann constant, T is the temperature, and $\mathcal{IS}$ (in mol/L) is the ionic strength.

The bonded interaction between two beads is described by a harmonic potential [2]:

$$U_{\mathrm{str}}(r_{ij}) = \frac{k_{\mathrm{str}}}{2}(r_{ij} - r_0)^2 \tag{S5}$$

where k_{str} is the spring constant and $r_{ij,0}$ is a reference distance.

Here, we introduced additional non-bonded terms. For beads representing H-bonded pairs, we added a stretching potential in the form of Equation (S5) with constant k_{bp} was Moreover, a harmonic bending potential between three consecutive beads in a strand was introduced to tune the stiffness of NA chains:

$$U_{\mathrm{bend}}(r) = \frac{k_{\mathrm{b}}}{2}(\theta - \theta_0)^2 \tag{S6}$$

where k_{b} is the strength constant and θ_0 is a reference angle between the three beads.

Model parameters The specific parameters for amino acids and nucleotides entering Equations (S1)-(S6) were taken from Refs. [3] and [4]. In particular, for the short-range non-bonded potential of amino acids we used values based on the Urry hydropathy scale, derived from experimental data on heat-induced conformational compaction of host-guest polypentapeptides, which were found to provide a good description of the phase separation of disordered proteins [4]. For nucleotides, we used the hydropathy scale proposed by Kapcha and Rossky (KR scale) [6] for proteins, that is based on the atomic charges in the OPLS all-atom force field [7]. The hydropathy parameters for DNA nucleotides were calculated according to the procedure outlined in Ref. [3] for RNA. For adenosine, cytosine and guanine, replacement of an OH by a hydrogen atom in ribose yields slightly larger hydrophobicity parameters; for thymine there is an additional contribution due to replacement of a methyl group in uracil by a hydrogen atom. The parameters of the U_{HPS} potential for all beads are listed in Table S1, along with the charge numbers Z_i. The parameter ϵ in Equation (S1) was set to be constant across all pairs and equal to 0.2 kcal/mol [3], whereas the values of σ_{ij} and λ_{ij} for beads of different types were computed as the arithmetic means $\sigma_{ij} = (\sigma_i + \sigma_j)/2$ and $\lambda_{ij} = (\lambda_i + \lambda_j)/2$, where a single subscript is used for interactions between beads of the same type. For the dielectric constant ϵ of water in Equation (S4), temperature-dependent values were used [8], ranging from 87.7 (at T=273.15 K) to 55.7 (at T= 363.15 K) [9].

The total non-bonded potential was truncated at a cutoff distance r_c and shifted in such a way that $U_{\mathrm{HPS}}(r_c) + U_{\mathrm{DH}}(r_c) = 0$. The value of r_{c} was taken equal to the distance at which the non-bonded interaction potential between two adenine beads, which have the strongest interaction, is

equal to 0.005 kcal/mol, in line with Ref. 3. For both peptides and DNA filaments, a stretching constant k_{str} =2.4 kcal/(mol Å^2) was used in Equation (S5), together with a reference distance r_0 equal to 3.8 Å for amino acids and to 5 Å for NTs [3, 4]. Moreover, for the stretching potential between base-paired NTs we used k_{bp} = 40 kcal/(mol Å^2), with a center-to-center distance equal to the arithmetic mean of their diameters, which is comparable to the average distances between the centers of mass of the NTs in B-DNA [10]. In the bending potential, Equation (S6), considering that the persistence length of dsDNA ($\sim$ 50 nm at 10 mM Na^+) is over 50 times greater than that of ssDNA ($\sim$ 0.75 nm at 10 mM Na^+) [11], we assumed k_{b} =40 kcal/(mol rad^2) and $\theta_0 = \pi$. To check the effect of flexibility, additional simulations were performed using a bending constant k_{b} = 2 kcal/(mol rad^2) for ssDNA and for dsDNA.

For PNA units, we used the same model and the same parameters as for DNA, with the only difference that charges were switched off. PolyP was modeled as a chain of soft beads with a reference distance of 3 Å, a diameter equal to 6 Å, a charge of -1 e and a hydropathy parameter equal to 0. A constant $k_{\mathbf{str}}$ = 1 kcal/(mol Å^2) in the stretching potential, Equation (S5), was assumed, which yields a persistence length, calculated from the decay of the autocorrelation function of bond vectors, similar to that of ssDNA.

Table S1: Charge numbers and parameters of the non-bonded short-range potential U_{PS} for amino acids and nucleotides, used in the CG-MD simulations.

AA/NT	Z_i	σ_i (Å)	λ_i
ALA	0.0	5.04	0.60
ARG	1.0	6.56	0.56
ASN	0.0	5.68	0.59
ASP	-1.0	5.58	0.29
CYS	0.0	5.48	0.65
GLN	0.0	6.02	0.56
GLU	-1.0	5.92	0.00
GLY	0.0	4.5	0.57
HIS	0.5	6.08	0.76
ILE	0.0	6.18	0.71
LEU	0.0	6.18	0.72
LYS	1.0	6.36	0.38
MET	0.0	6.18	0.68
PHE	0.0	6.36	0.82
PRO	0.0	5.56	0.76
SER	0.0	5.18	0.59
THR	0.0	5.62	0.59
TRP	0.0	6.78	1.00
TYR	0.0	6.46	0.90
VAL	0.0	5.86	0.66
ADE	-1.0	8.44	0.08
CYT	-1.0	8.22	0.11
GUA	-1.0	8.51	-0.05
THY	-1.0	8.17	0.19

Simulation setup Phase separation was investigated using the slab method [2]. Initially, straight chains were randomly placed inside a cubic box with periodic boundary conditions in all three directions. Energy minimization was performed to remove steric clashes among the chains. Then, the system was equilibrated in the NPT ensemble (100 ns) at a temperature of 150 K and a pressure of 1 bar. During this process, the box was compressed until reaching

the equilibrium size. Subsequently, the box was elongated in one dimension (Z axis) to obtain the desired concentration and the system was brought to the target temperature by a 100 ns trajectory in the NVT ensemble. Finally, a 5 μs simulation in the NVT ensemble was carried out, which includes 1 μs of equilibration followed by 4 μs of production. No major changes were observed by increasing the length of the production run.

To build the phase diagrams, several simulations were run at different temperatures and ionic strengths. Samples of $\sim$ 5000 beads (around 200 peptides and a number of polyanions so as to neutralize the positive charges) were used, at 120 mM polycation concentration. We performed also a few simulations at the same peptide concentration used in experiments (i.e. $C_{pL22} = 300\,\mu$M), taking the same number of polycations and polyanions as in the more concentrated samples and elongating the Z axis of the box in the slab method, to reach the target concentration. To check for finite-size effects, additional simulations were performed on some of the systems, doubling the size of the sample ($\sim$ 10000 beads), but no significant changes were observed.

For specific state points, we performed bulk simulations of larger systems ($\sim$ 25000 beads), using periodic boundary conditions in the NPT ensemble with zero external pressure [12]. The simulations started from randomly placed and oriented chains, at a density equal to the density of the clusters obtained by slab simulations at the same temperature and ionic strength.

All simulations were carried out using the LAMMPS package [13]. The equations of motion were integrated with the velocity Verlet algorithm using a timestep of 10 fs. Temperature was controlled by a Langevin thermostat [14] with a damping parameter of 100000 fs; for NPT simulations, a Nose-Hoover barostat [15] with a damping parameter of 1000 fs was applied. Pair interaction potentials were provided in tabular form.

Analysis of trajectories Trajectories were analyzed using home-developed *python* scripts and routines from the package *MDAnalysis*[16]. Phase separation was monitored through the distribution of cluster sizes, calculated over the last 4 μs of the trajectory. Phase separation was assumed when the majority of peptides and DNA oligomers (over 50 %) was contained in a large persistent cluster, in a bath of small aggregates, each made of very few chains. The opposite situation, with chains distributed in small clusters, was identified as a single phase. In a few cases, an intermediate behavior was also observed, with one or two relatively big clusters, containing 20 − 30 % of chains, with a short lifetime, together with very small aggregates. For the structural characterization of the condensates we calculated the gyration tensor of the clusters and of the peptide and NA chains, the Radial Distribution Function (RDF) of the beads and the $\langle P_2 \rangle$ order parameter. The mobility of the chains was characterized by the Mean Square Displacement (MSD) of the center of mass of the chains and by the number of exchanges of peptides/DNA with outside the clusters. To improve sampling, RDFs, translational diffusion coefficients, $\langle P_2 \rangle$ order parameters and average number of contacts were calculated from bulk simulations.

Gyration tensor The gyration tensor of a chain or of a cluster was calculated as

$$\mathbf{T}_{\mathrm{g}} = \frac{1}{N_{\mathrm{b}}} \sum_{i=1}^{N_b} \left(\vec{r}_i \otimes \vec{r}_i - \vec{r}_{\mathrm{CM}} \otimes \vec{r}_{\mathrm{CM}} \right) \tag{S7}$$

where N_{b} is the number of beads, $\vec{r}_i$ is the vector position of the ith bead and $\vec{r}_{\mathrm{CM}}$ is the vector position of the center of mass of the chain/cluster.

Orientational order parameter The orientational order was quantified through the order parameter $\langle P_2 \rangle$, i.e. the average value of the second Legendre polynomial, having as its argument the cosine OF the angle between the director $\hat{n}$ (a unit vector parallel to the C_∞ symmetry axis of the system) and the long axes of the aligning units. Considering that in our systems the orientational order is determined by the dsDNA chains, the director was calculated by diagonalizing

the quadrupolar ordering tensor: [17]

$$\mathbf{Q} = \frac{1}{2N_{\mathrm{DNA}}} \sum_{i=1}^{N_{\mathrm{DNA}}} \left(3\,\hat{u}_i \otimes \hat{u}_i - \mathbf{I}_3\right) \tag{S8}$$

where N_{DNA} is the number of DNA chains, $\mathbf{I}_3$ is the identity matrix, and $\hat{u}_i$ is a unit vector parallel to the alignment axis of the i-th chain, which was taken parallel to the major eigenvector of the gyration tensor of the chain. The director $\hat{n}$ is the major eigenvector of the $\mathbf{Q}$ tensor and the corresponding eigenvalue is the order parameter of the DNA chains, $\langle P_2 \rangle_{\mathrm{DNA}}$. The phase was labeled as LC if $\langle P_2 \rangle_{\mathrm{DNA}} \gtrsim 0.8$.

For peptides, we took the chain bonds as the aligning units; thus the order parameter was calculated as:

$$\langle P_2 \rangle_{\mathrm{pep}} = \frac{1}{2N} \sum_{i=1}^{N} \left(3\,\hat{u}_i \cdot \hat{n} - 1\right) \tag{S9}$$

where N is equal to the product of the number of peptide chains times the number of bonds in a chain and $\hat{\mathbf{u}}_i$ is a unit vector parallel to a peptide chain bond.

Definition of clusters and calculation of chain densities For the definition of clusters we used a bead-to-bead distance metric, i.e., a chain belongs to a cluster if it has at least one bead within a distance d_c from any other chain of the cluster. Based on the radial distribution function calculated for the various species, $d_{\mathrm{c}} = 15$ Å was assumed.

In the presence of phase separation, the dense phase concentration was calculated as the ratio between the mass of the chains contained in the largest cluster and the volume of the cluster. This was estimated as the volume of a sphere with a radius equal to the average gyration radius of the cluster. The density of the dilute phase was then computed from the mass of the remaining polymer chains and from the volume of the box outside the cluster.

Average number of contacts To calculate the average number of contacts of amino acids with nucleotides, we counted the NT beads having their centers within a cut-off radius (r_{c}) from the centers of AA beads. Based on the values of σ_i (Table S1), $r_{\mathrm{c}} = 9$ Å, was assumed.

Dynamic quantities The translational diffusion coefficients of chains were determined by linear fitting of the mean square displacements (MSDs) of the centers of mass of single chains *vs* lag time in the diffusion regime. Analogously, for liquid-crystalline systems, we calculated the diffusion coefficients along the director and perpendicular to it. For the calculation of the number of exchanges in/out of a cluster, a chain was assumed to be in the cluster unless all its beads were outside.

oxDNA simulations

oxDNA CG simulations of several oligonucleotides were performed using the sequence-specific *DNA2* parameterization [18], which accounts for base-specific stacking and hydrogen bonding, and allows for tuning the monovalent salt concentration. Initial conformations were built using *oxView* [19] and subsequently subjected to energy minimization *via* 1000 steps of *Monte Carlo* sampling. Before the actual production simulation, an additional MD relaxation phase of 10^7 steps using a *Langevin thermostat* with a lowered diffusion coefficient ($D = 0.5$ *SU*) and a small integration time step ($dt = 0.0005$ *SU*) is performed. Starting from the relaxed configuration, the production MD phase in the NVT ensemble (*Andersen thermostat*, $D = 2.5$ SU) lasted for ~ 1.8 μs ($dt = 0.003$ SU). Configurations were printed every 10^4 steps only after an initial equilibration period of 10^6 steps. All simulation phases described are performed at 25°C and at 50 mM salt concentration. Trajectories were aligned and analyzed with the aid of the *oxDNA Analysis Tools* [19] package.

Quantification of chain flexibility

The flexibility of DNA oligomers was estimated using a normalized Root Mean Square Deviation ($RMSD_n$) to account for the intrinsic length dependence of RMSD [20] in short and highly charged systems: To compute the RMSD time series, the centroid structures and configurational ensembles of all oligonucleotides were obtained by *oxDNA* simulations, as described above. For the G-quadruplex sequence in Fig. S11, the NMR ensemble in PDB 1AXV was used and the centroid structure and unweighted deviations were calculated from the 20 configurations depleted from hydrogens and K^+. Instead, the configurations of polyP were sampled by a 100 ns long all-atom MD trajectory of a fully deprotonated 20mer in water [21], in the presence of 22 Na^+ ions. Standard parameters of the OPLS-AA force field [22] were used for polyphosphate and Na^+ ions, together with the TIP3P water model. Simulations were performed using GROMACS (www.gromacs.org).

Given a set of configurations of a flexible chain made of N beads, the RMSD of the k-th configuration with respect to a reference structure can be defined as:

$$RMSD^{(k)} \doteq \sqrt{\frac{1}{N}\sum_{i=1}^{N}\left(\vec{x}_i^{(k)} - \vec{x}_i^{(m)}\right)^2} \tag{S10}$$

where $\vec{x}_i^{(k)}$ and $\vec{x}_i^{(m)}$ are the coordinates of the i-th bead in the k-th configuration and in the reference structure, respectively. In our calculations, we took as a reference the centroid of the structures comprised in the configuration set. Because the time-averaged $RMSD = \left\langle RMSD^{(k)}\right\rangle_k$ scales linearly with the length of the DNA strands (see Fig. S10), we implemented the simple normalization:

$$RMSD_n \doteq \frac{RMSD}{\mathcal{N}} \tag{S11}$$

where $\mathcal{N}$ is the number of monomers of the ONT, i.e., single nucleotides for ssDNA, base pairs for dsDNA, quadruplets for G-quadruplexes, and the proper combination for partially hybridized structures (in analogy with the definition of $\mathcal{L}_c$).

Calculation of the average surface electrostatic potential

To calculate the average electrostatic potential generated on the surface of a polyanion by its charge distribution, we used atomistic representations of the systems, with centroid structures of the oligomers, obtained for each system as described in the previous Section (CG coordinates from *oxDNA* configurations were transformed into PDB using *tacoxDNA*, tacoxdna.sissa.it). Starting from the atomic coordinates, the partial charges and atomic radii defined according to the AMBER03 force field, were obtained using the APBS-PDB2PQR software suite [23]. The molecular surface was defined by a finite set of points, determined over a uniform grid in a rectangular box containing the molecule. The Coulomb potential ϕ_e was evaluated at each point, assuming a relative dielectric constant $\epsilon = 2$ inside the polyanions. The average surface potential Φ_e was calculated over the grid by discrete summation.

$$\Phi_e \doteq \frac{1}{S}\int_S \phi_e \, d\sigma \approx \frac{\sum_i \phi_e(\mathbf{r}_i)\Delta S_i}{\sum_i \Delta S_i} \tag{S12}$$

where S is the surface area and ΔS_i are surface elements. The defined Φ_e bears an intrinsic dependence on the polymer length, arising from the long-range nature of the Coulomb potential and from the combinatorial growth of the grid points where the potential is evaluated. Therefore, in a similar fashion to the definition of the $RMSD_n$, we normalize the average surface potential Φ_e as:

$$\widetilde{\Phi}_e \doteq \frac{\Phi_e}{\ln \mathcal{N}} \tag{S13}$$

Normalization of the electrostatic potential Consider a linear chain of $\mathcal{N}$ identical point charges q, where $\mathcal{N}$ is an odd integer and the inter-charge distance is l. The total potential experienced by a probe charge that explores $\mathcal{N}$ discrete positions, each located at a distance $d \ll l$ from a respective point charge, can be expressed as:

$$\Phi_e = \frac{\mathcal{N}A}{d} + (\mathcal{N}-1)\frac{A}{l} + 2\sum_{i=1}^{\frac{\mathcal{N}-1}{2}} \frac{2A}{il}\left(\frac{\mathcal{N}}{2} - i\right) + 2\sum_{i=1}^{\frac{\mathcal{N}-1}{2}} \frac{A}{il} \tag{S14}$$

where $A = q/4\pi\epsilon_0$, with ϵ_0 the vacuum permittivity. In the limit $\mathcal{N} \to \infty$ this can be approximated by:

$$\frac{\Phi_e\, l}{A\mathcal{N}} \simeq \alpha + 2\,(\gamma - \ln 2) - 1 + 2\,\ln\mathcal{N} + 2\,\frac{\ln\mathcal{N}}{\mathcal{N}} + \mathcal{O}\left(\frac{\ln\mathcal{N}}{\mathcal{N}}\right) \tag{S15}$$

with $\alpha \doteq l/d$ and γ the Euler-Mascheroni constant. Thus the scaling of the average surface electrostatic potential $\Phi_e/\mathcal{N} \sim \ln\mathcal{N}$ and the normalization in Equation 4.

Experimental methods

Determination of phase diagrams In our study, we consider mixtures with ratio of positive-to-negative polyelectrolyte charges equal to one. Thus, for different ONTs (where the length or the number of charges-per-monomer change), the stoichiometric composition of the mixture changes accordingly, at fixed pL22 concentration (if not stated otherwise, $C_{pL22} = 300\,\mu$M).

A 3 % Bovine Serum Albumin (BSA, Sigma-Aldrich) solution was used to coat plastic well plates (Amine Treated 96 well plates) by adding $\sim$ 80 μL of solution for about 20 minutes, removing it and washing twice with $\sim 100\,\mu L$ Milli-Q water for about 10 minutes.

Coacervate samples were prepared in a total volume of 10 μL in 200 μL Eppendorf tubes, mixing the components in this given order: Milli-Q water, Tris-HCl buffer, NaCl, pL22 and polyanions. For all the samples, the ionic strength of the mixture is estimated as the molar concentration of added salt plus the 15 mM brought by the 20 mM Tris-HCl buffer. Mixtures were prepared at room temperature and after the final addition of polyanions the solution was mixed by gentle pipetting. After a few seconds, coacervate samples were transferred to the center of well plates previously treated with BSA. To avoid contamination and evaporation of the samples, each well was covered with $\sim$ 100 μL mineral oil (M5904, Sigma-Aldrich) and a plastic PCR film was applied on the plate.

Samples were imaged using a Nikon TE200 microscope, equipped with a Nikon DS-5M CCD camera and temperature-controlled chamber. Before imaging, coacervate droplets were let sediment at 24 °C for about 30 minutes. Samples were then heated up to 90 °C at 1.5 °C/min and, after about 10 minutes at 90 °C, were imaged again. Subsequently, samples were cooled down at 1.5 °C/min with 5 °C steps and phase state was annotated at each temperature step after a few minutes of equilibration.At the end of the cooling ramp, samples were imaged at 20 °C and then at $10\,°C$ after a final cooling step at 1.5 °C/min. All temperature-salt phase diagrams reported in the main text refer to data acquired during the cooling ramp.

Determination of local concentration To estimate the DNA concentration in the dense phase (C_d) for a given stoichiometry (C_s), the volume fraction of the dense phase (φ) and the concentration of DNA in the supernatant (C_l) were measured; C_d can be then extracted from mass conservation:

$$C_d = \frac{C_s - C_l}{\varphi} - C_l \tag{S16}$$

To estimate the volume fraction of the dense phase, fluorescently labeled samples were prepared and imaged as described in Materials and Methods, Mobility. A z-stack with equal spacing between slices ($z_{gap} \sim 300$ nm) of a large field of view ($\sim 34116\,\mu\text{m}^2$) of the sample was acquired.

After thresholding of the whole stack using the ImageJ software, the volume fraction φ is simply computed as:

$$\varphi = \frac{z_{gap}}{h} \sum_s A_f^{(s)} \tag{S17}$$

where $h = 1000\,\mu$m is the height of the sandwiched sample and $A_f^{(s)}$ is the area fraction of pixels belonging to the coacervate phase in the slice s.

To estimate the DNA supernatant concentrations, samples were prepared in small Eppendorf tubes and droplets were let sediment at ambient temperature for at least one hour; subsequently, the Eppendorf tubes were centrifuged for 1 minute and few microliters were carefully removed from the supernatant phase. DNA concentrations were then measured *via* standard UV-absorbance at 260 nm, $C_l = A_{260}/(\epsilon_{260}\, l)$, where l is the variable optical path of the micro-spectrometer and ϵ_{260} is the sequence-specific extinction coefficient of the DNA sequence.

Measurements of both φ and C_l were performed in triplicates and the error on C_d was estimated from the error propagation of Equation (S16), where: (i) the error on C_s is computed from the pipetting error due to stock solutions dilutions; (ii) the error on C_l is given from the standard deviation of 3 independent UV-absorbance measurements; (iii) the error on φ is given from the standard deviation of 3 z-stacks taken from different field of views.

FRAP measurements and analysis FRAP measurements were performed exploiting a laser scanning Confocal Microscope (Leica Stellaris 8) equipped with a 63× ($NA = 1.40$) oil immersion objective (HC PL APO CS2) and a sCMOS camera Leica DFC9000 GTC. The imaging system is operated using the LASX (LEICA) Software.

Photobleaching is achieved by using a 495 nm laser at maximum output for 2-5 cycles for the FAM fluorophore or a 530 nm laser at maximum output for 5-10 cycles for TAMRA (each cycle lasting approximately 500 ms). For a given droplet, bleaching is performed on a circular region of interest (ROI) of diameter of the order of 1 μm. For each bleached ROI, a sequence of 5 frames is recorded prior to bleaching (typical duration of 5 s) and one of 100-200 frames immediately after bleaching (typical duration of 1-2 minutes). To monitor photobleaching during the acquisition, only droplets close to at least one other droplet, chosen as a reference, are considered.

For each FRAP measurement, the pre- and post-bleaching raw data series are analyzed without further image pre-processing. Using Image-J, a circular ROI corresponding to the bleach spot is drawn on the first frame of the post-bleaching series and the average intensity $I_{post}(t)$ is measured; using the same exact ROI, the pre-bleaching average intensity $I_{pre}(t)$ is extracted. Moreover, a round ROI of variable size is drawn outside any droplet to quantify the average background intensity $I_{bkg}(t)$, while one is drawn on a reference unbleached droplet to estimate the unwanted photobleaching $I_{blch}(t)$.

The $I_{pre}(t)$, $I_{post}(t)$ and $I_{blch}(t)$ signals are first corrected by subtracting the background intensity: $\tilde{I}_i(t) = I_i(t) - I_{bkg}(t)$. Then, the post-bleaching intensity is normalized with respect to the unwanted photobleaching, $\tilde{I}^*_{post}(t) = \tilde{I}_{post}(t) \,/\, \tilde{I}_{blch}(t)$, and the same is done for the time-averaged pre-bleaching intensity: $\tilde{I}^*_{pre} = \langle \tilde{I}_{pre}(t) \rangle \,/\, \langle \tilde{I}_{blch}(t) \rangle$. The normalized integrated time-dependent concentration after photobleaching is then defined as:

$$C^*(t) = \frac{\tilde{I}^*_{post}(t) - \tilde{I}^*_{post}(0)}{\tilde{I}^*_{pre} - \tilde{I}^*_{post}(0)} \tag{S18}$$

The time evolution of $C^*(t)$ is modeled with a simple exponential recovery:

$$C^*(t) = A\left(1 - e^{-t/\tau}\right) \tag{S19}$$

where A is the fractional recovery and $\tau = R^2_{bleach} \,/ D_{app}$, with D_{app} being the apparent diffusion coefficient. Fitting the above equation to the experimental concentration profile allows for the estimation of D_{app} and A as the best-fitting parameters.

Raman spectra analysis Single-point Raman spectra were pre-processed using the *RamanSPy python* package [24]. For both dry and coacervate spectra, a custom pipeline is built to perform: (i) cropping in the spectral region [600, 1800] cm^{-1} for dry samples or [1150, 1750] cm^{-1} for coacervate samples; (ii) cosmic rays removal using the Whitaker-Hayes algorithm (default parameters); (iii) spectral denoising using a Savitzky-Golay filter (window 15, polynomial order 2); (iv) baseline correction based on asymmetric least squares (smoothing constant 1000000, differential order 2, max iterations 50); (v) vector normalization. Spectra were then averaged and deviations computed as standard errors.

For pre-processing and analysis of Raman maps, the Orange software (version 3.36.1) was used and the following pipeline is implemented: (i) cropping in the spectral region [1150, 1750] cm^{-1}; (ii) rubber band baseline correction; (iii) spectral denoising using a Savitzky-Golay filter (window 11, polynomial order 2), followed by gaussian smoothing (one standard deviation) and PCA denoising with 4 components; (iv) vector normalization.

Table S2: All the DNA/PNA oligomers employed in this study: abbreviations, primary structures and secondary structures in dot-bracket notation; the & sign indicates ligation of two strands, while bold sequences refer to PNA traits.

Abbreviation	Primary structure
ssPNA	5'-**GGACGACTTG**-3'
ssDNA-10	5'-GGACGACTTG-3'
ssDNA-20	5'-GTTAAAGTGCCAAGTCGTCC-3'
ssDNA-40	5'-GCATATCATCGGTACACATCATCTCGGCAGGGGTCAGTTA-3'
ssDNA-80	5'-CACAACACCCACAACCAAAAACAACAAACCACACCCCAAC<br>CCACACAAACACACCCCAACAACCACCACAACCAACAACC-3'
hdsDNA	5'-GTTAAAGTGCCAAGTCGTCC-3'<br>5'-GGACGACTTG-3'
hp5DNA	5'-TGCGAGAGATATCTCGATCC-3'
dsDNA-10	5'-GGACGACTTG-3'<br>5'-CAAGTCGTCC-3'
dsDNA-20	5'-GTTAAAGTGCCAAGTCGTCC-3'<br>5'-GGACGACTTGGCACTTTAAC-3'
dsDNA-40	5'-GCATATCATCGGTACACATCATCTCGGCAGGGGTCAGTTA-3'<br>5'-TAACTGACCCCTGCCGAGATGATGTGTACCGATGATATGC-3'
PNA-10 hybrid	5'-**GGACGACTTG**-3'<br>5'-CAAGTCGTCC-3'

Abbreviation	Secondary structure
ssPNA	None
ssDNA-10	None
ssDNA-20	None
ssDNA-40	None
ssDNA-80	None
hdsDNA	..........((((((((((&))))))))))
hp5DNA	..(((((....)))))....
dsDNA-10	((((((((((&))))))))))
dsDNA-20	((((((((((((((((((((&))))))))))))))))))))
dsDNA-40	((&<br>&))
PNA-10 hybrid	**((((((((((**&))))))))))

Table S3: pL22 sequence and principal physicochemical properties. Positively charged residues at neutral pH are highlighted in red, negative ones in blue; “GRAVY” stands for ”Grand Average of Hydropathy, “SCD” for Sequence Charge Decoration.

Sequence	LKRVLPRARGRADIIKKR
Molecular weight	2146.6 Da
Net charge at pH 6-8	+ 7.0
Theoretical pI	pH 12.58
SCD	+ 2.8
GRAVY	−0.85

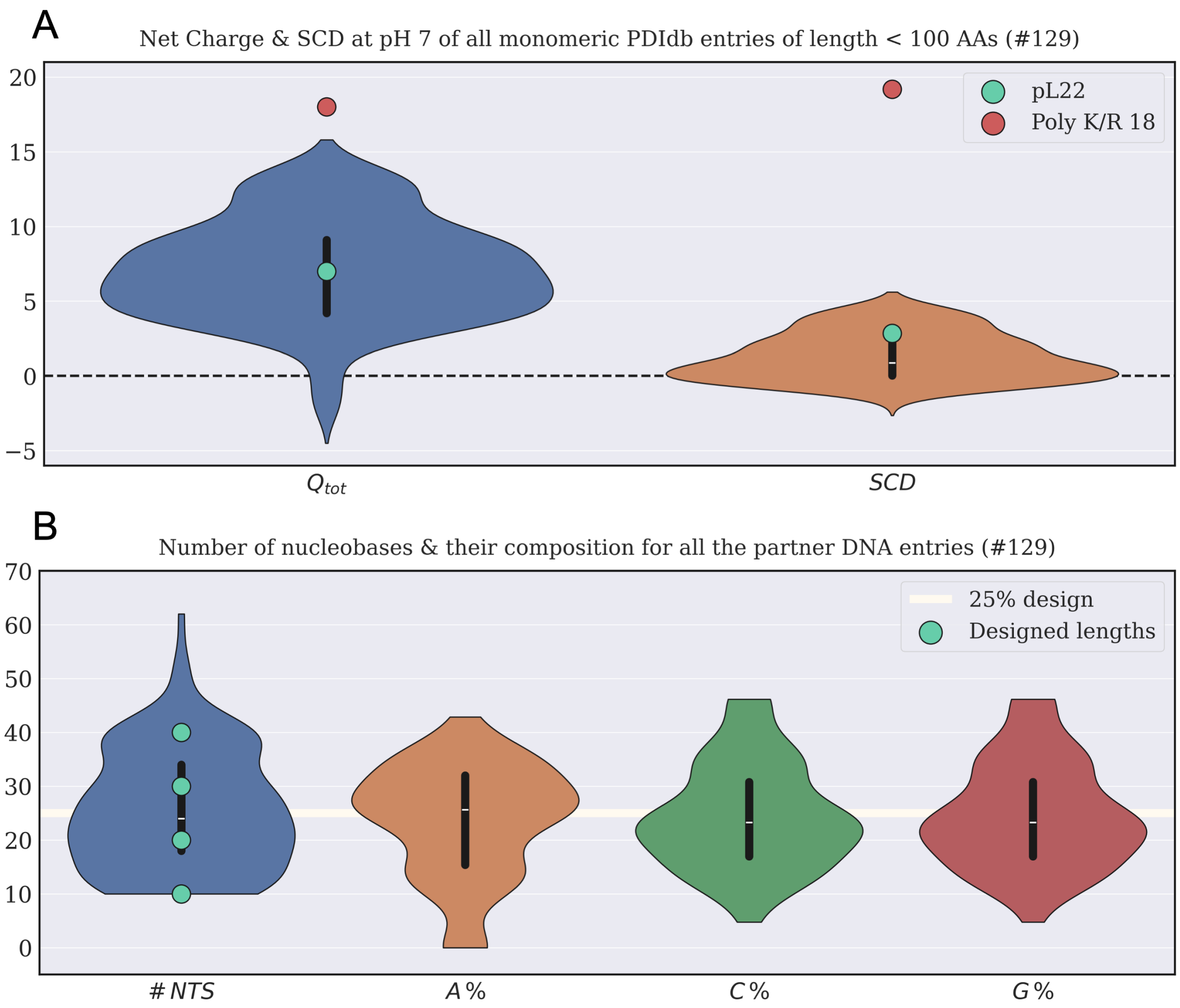

Figure S1: *Comparison between pL22, the designed DNA sequences and a subset of 129 entries from the* PDIdb *repository (http://melolab.org/pdidb/web/content/home), containing relevant structural information of protein-DNA complexes. The selected entries correspond to all the DNA-binding proteins with less than 100 AAs and lacking quaternary structure. A) Violin plots of net charge and Sequence Charge Decoration (SCD) of the proteins at pH 7.0 (R,K = +1; D,E = -1; H = +0.1). The green markers refer to the net charge and SCD of pL22, while the red ones to the same quantities for a Poly-L-lysine or Poly-L-arginine of the same length. B) Violin plots of the number of nucleotides and nucleobases composition in the DNA sequences corresponding to panel A; green markers show the probed DNA lengths in this study.*

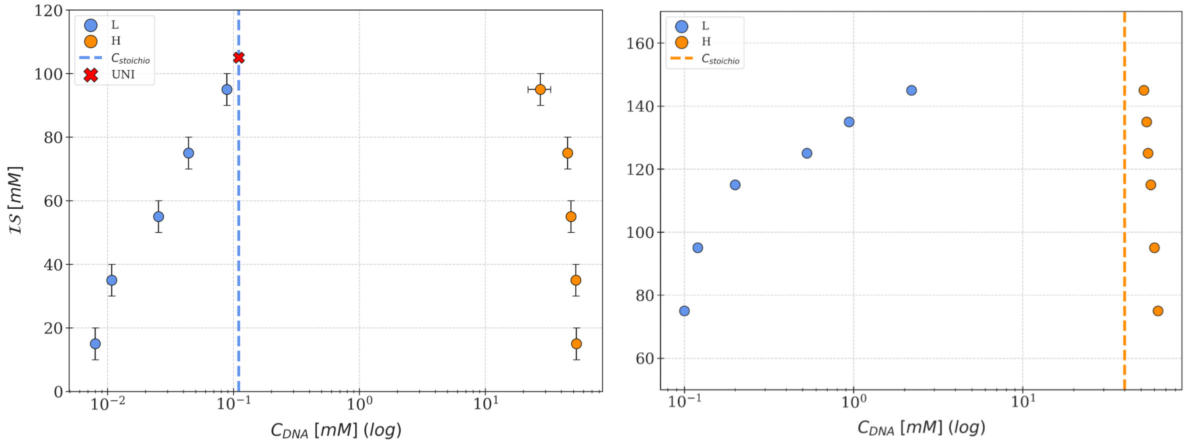

Figure S2: *Experimental (left) and simulated (right) phase diagrams in the DNA concentration-ionic strength of the solution plane (x-axis, log scale, and y-axis, lin scale, respectively). "UNI" denotes no demixing, while "L" and "R" refer to the "left" and "right" branches of the bell-shaped diagram. Concentrations of the dense phase (R) are estimated from the concentrations of the supernatant phase (L), scanning the line at constant concentration "$C_{stoichio}$" of DNA and pL22.*

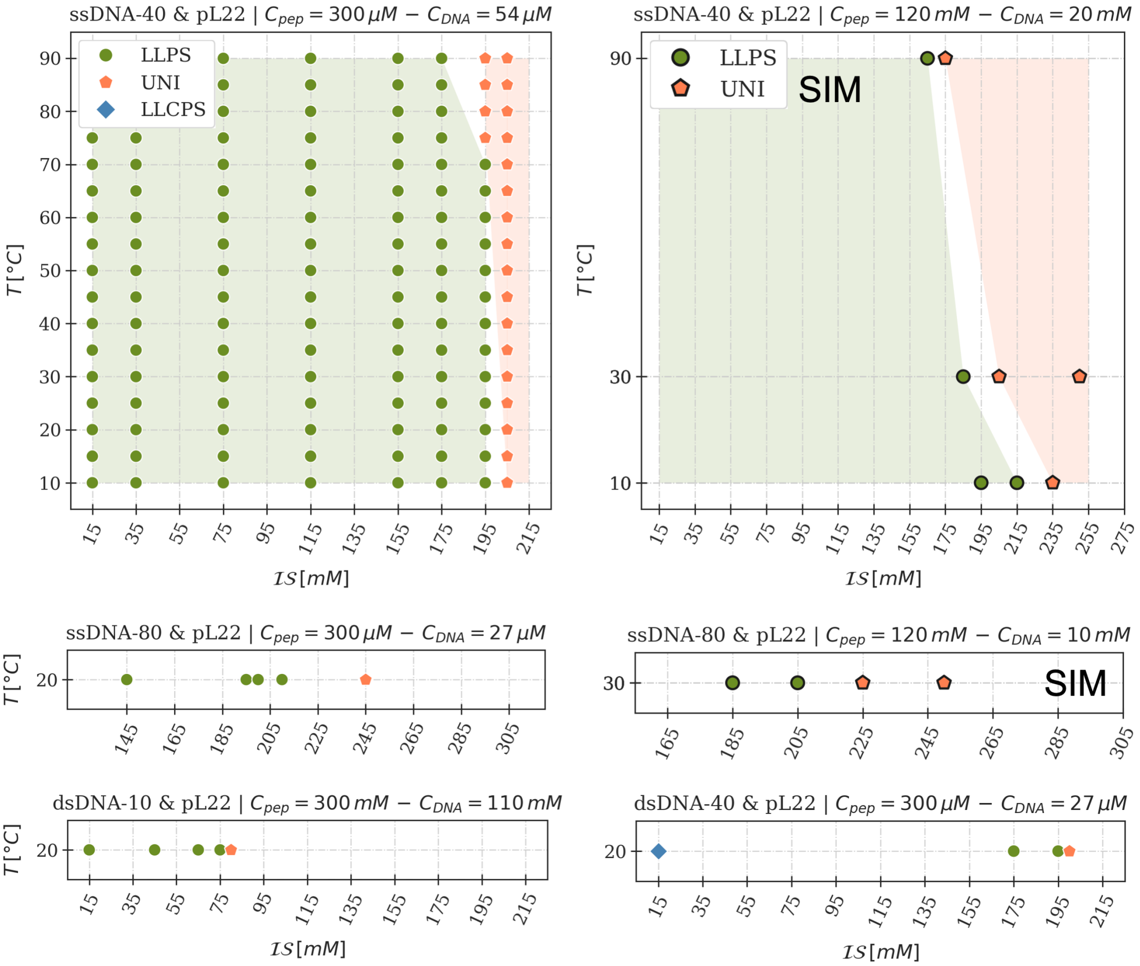

Figure S3: *Experimental and simulated (marked by "SIM") phase diagrams of mixtures of pL22 with ssDNA-40 (first row, left & right), ssDNA-80 (second row, left & right), dsDNA-10 (third row, left) and dsDNA-40 (third row, right). For ssDNA-40 the full $T - \mathcal{IS}$ phase diagrams are shown, while for the other oligonucleotides the phase behavior was annotated only at $T = 20\,°C$ (or $T = 30\,°C$ for simulated ssDNA-80) after temperature annealing. Phases are denoted by the color and shape of markers and shaded regions: monophasic (orange pentagons), isotropic biphasic (green full circles) and liquid-crystalline biphasic (blue diamonds).*

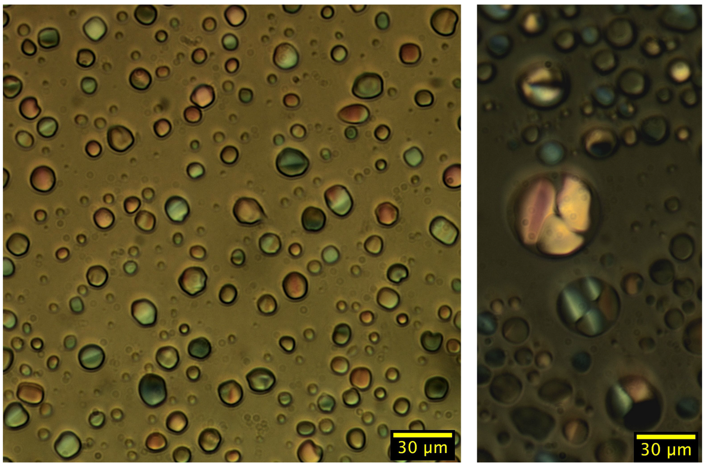

Figure S4: *Polarized optical microscopy images of dsDNA-20 and pL22 coacervates (C_{DNA}=55 μM & C_{pL22}=300 μM) at ambient temperature and $\mathcal{IS}$=95 mM (left) or $\mathcal{IS}$=15 mM (right). In both cases, birefringent textures indicate liquid-crystalline ordering.*

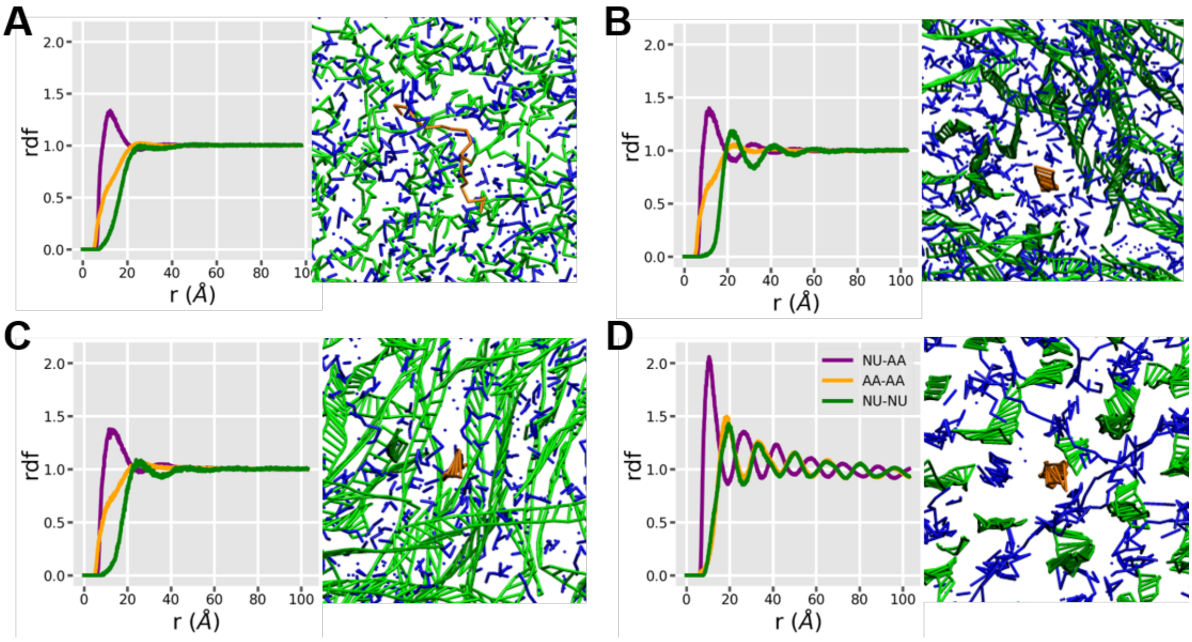

Figure S5: *Radial Distribution Functions (rdf) inside coacervates (left) and corresponding illustrative snapshots (right) from bulk MD simulations at* T = 303 K *of: A) pL22/ssDNA-20, $\mathcal{IS}$=75 mM (isotropic); B) pL22/dsDNA-20, $\mathcal{IS}$=75 mM (LC); C) pL22/dsDNA-20, $\mathcal{IS}$=225 mM (isotropic); D) polyK-18/dsDNA $\mathcal{IS}$=75 mM (solid). The plots show the rdf calculated for amino acids belonging to different peptides (orange), for DNA nucleotides belonging to different ONTs (green), and for amino acids around DNA nucleotides (violet). In the snapshots, DNA chains are colored green, whereas blue is used for amino acids within 9 Å from DNA nucleotides; the DNA chain in the middle is highlighted in orange for reference. The rdfs in isotropic coacervates in C are only slightly more structured than those in A, both indicating direct contacts between amino acids and nucleotides, but no contacts between the nucleotides and scarce, yet not fully absent, contacts between peptides. The same holds for LC coacervates in B, but in this case the structure of the rdfs for DNA-DNA and peptide-DNA pairs provides clear evidence of two coordination shells. In D the order is even more pronounced and extends to longer range; moreover, there are no contacts between peptides, which can be attributed to their mutual electrostatic repulsion in the highly charged polyK.*

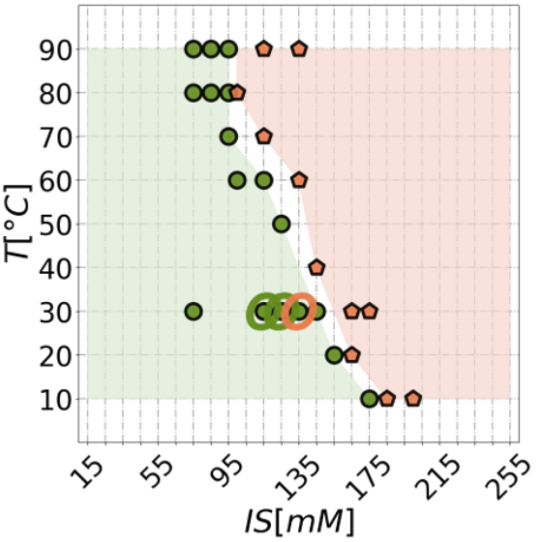

Figure S6: *Phase diagram of pL22/ssDNA-20 mixtures as a function of the temperature* T *and of the ionic strength* IS*, obtained from slab-MD simulations starting from* $C_{DNA} = 40$ *mM and* $C_{pL22} = 120$ *mM. The phase behavior is denoted by the color and shape of markers and shaded regions: monophasic (orange pentagons) and isotropic biphasic (green full circles). The orange/green empty circles show the results obtained using the same concentration used in experiments, namely* C_{DNA}^{sim}*=110* μ*M and* C_{pL22}^{sim}*=300* μ*M.*

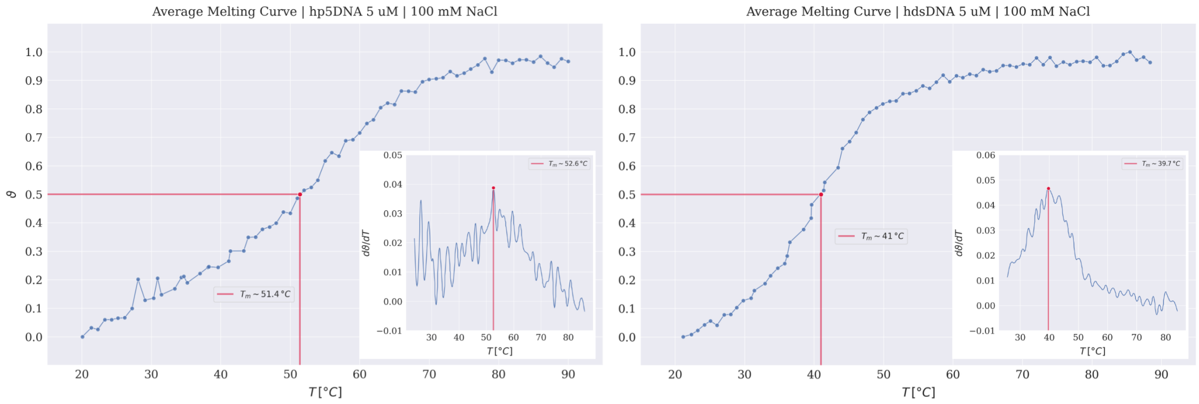

Figure S7: *Normalized absorbance melting curves of hp5DNA (left) and hdsDNA (right) at 5* μ*M in aqueous 100 mM NaCl solution. Absorbance spectra in the 210-320 nm range are collected and the raw signals around the 260 nm peak are then averaged and normalized. The value* ϑ*=0.5 determines a first estimate of the melting temperature (indicated by the red markers and lines), around 51* $^\circ C$ *for hp5DNA and 41* $^\circ C$ *for hdsDNA. The insets of the two curves show the numerical differentiation of the melting curves, upon smoothing and interpolation, with the maxima providing a more reliable estimate of the melting temperatures (53* $^\circ C$ *for hp5DNA and 40* $^\circ C$ *for hdsDNA) due to baseline removal.*

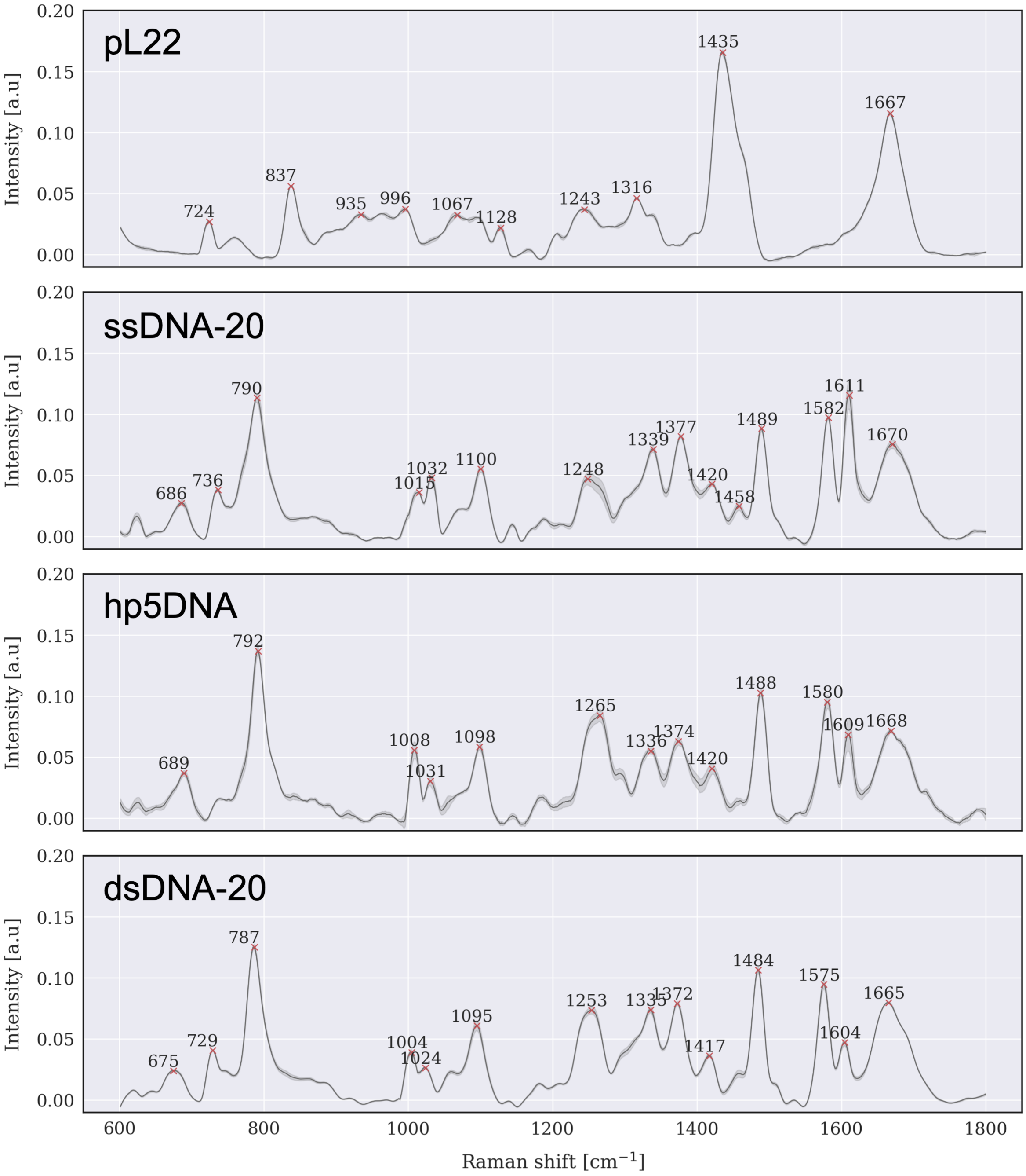

Figure S8: *Average Raman spectra and deviations for dry samples of pL22 (n =3), ssDNA-20 (n =8), hp5DNA (n =6) and dsDNA-20 (n =6). Raw data are are processed and normalized as described in the Supplementary Data text.*

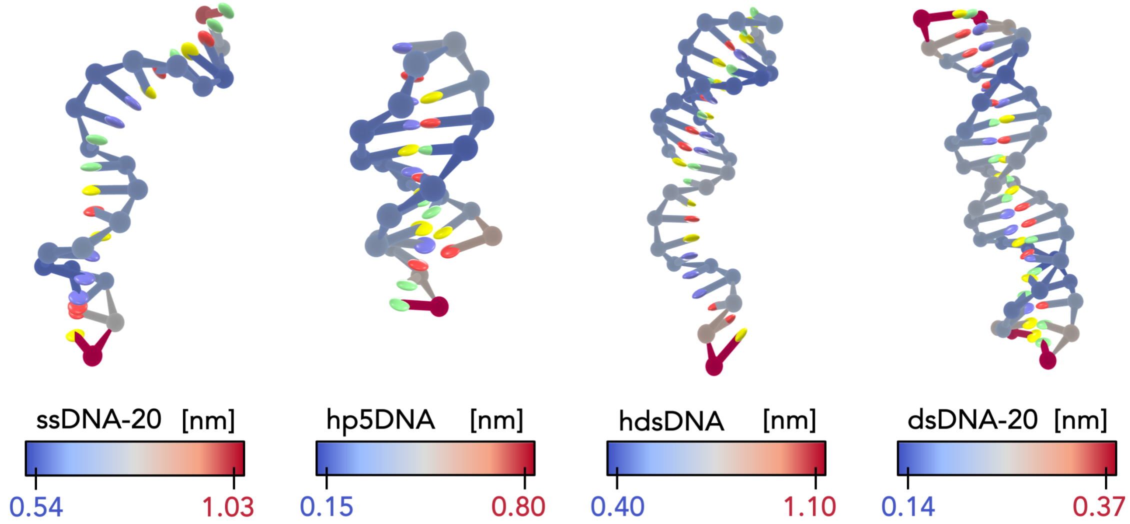

Figure S9: *oxDNA centroid structures (from letf to right: ssDNA-20, hp5DNA, hdsDNA and dsDNA-20) with a Root Mean Square Fluctuation (RMSF) overlay over the backbone (blue-red color mapping). The RMSF is computed as the time averaged per-residue RMSD over the course of whole production simulation.*

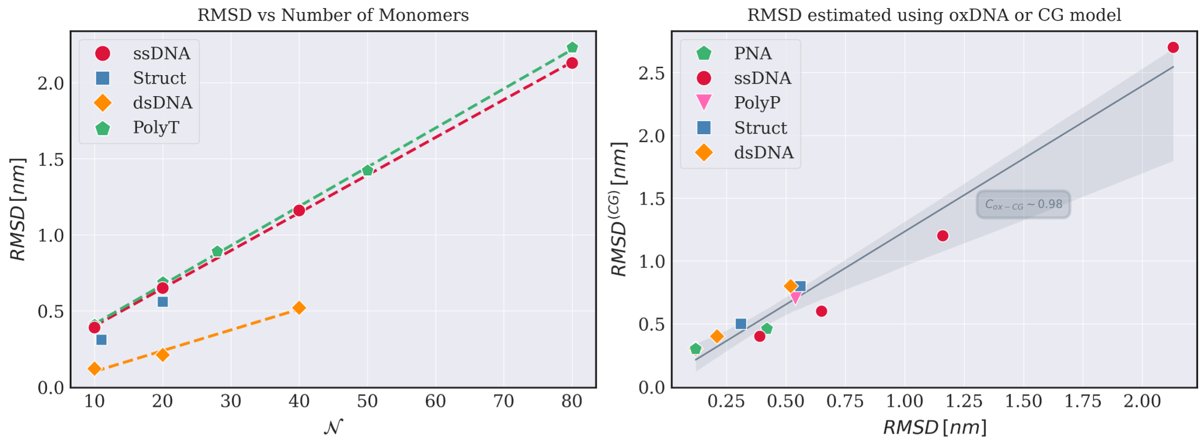

Figure S10: *Left panel: Root Mean Square Deviation (RMSD) dependence on the number of monomers of several DNA strands, as estimated from oxDNA simulations; along with the ss and ds sequences, hp5DNA and hdsDNA are shown, together with a series of Poly-Thymine (PolyT) of different lengths. The dotted line is a fit to a line, showing how the scaling of the RMSD is clearly linear both in ss, ds and homopolymeric sequences. Right panel: correlation plot between the RMSD values estimated from oxDNA and from the CG model employed in this study; a linear regression with the relative interval region is shown, together with the Pearson correlation coefficient.*

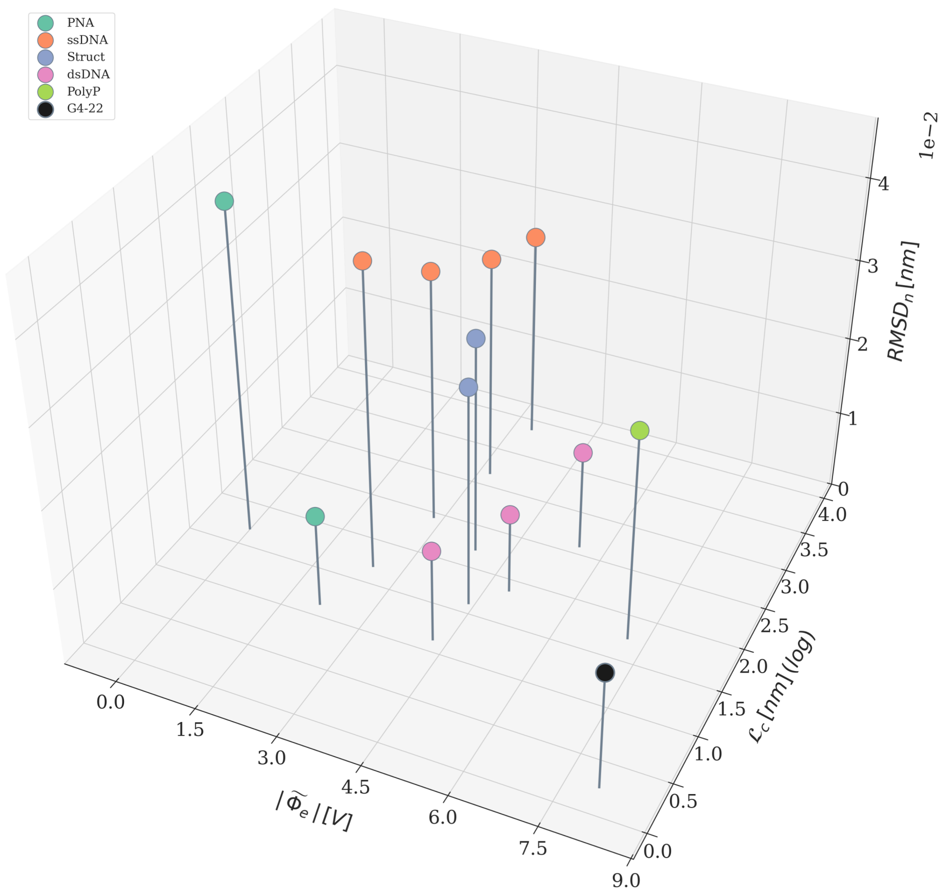

Figure S11: *Comparison of the ONTs employed in this study with a G-quadruplex of 22 nucleotides (G4-22) in the parameter space of contour length $\mathcal{L}_c$, normalized average surface electrostatic potential $|\widetilde{\Phi}_e|$ and normalized $RMSD_n$. The color-coding refers to the different classes of sequences, with G4-22 shown in black.*

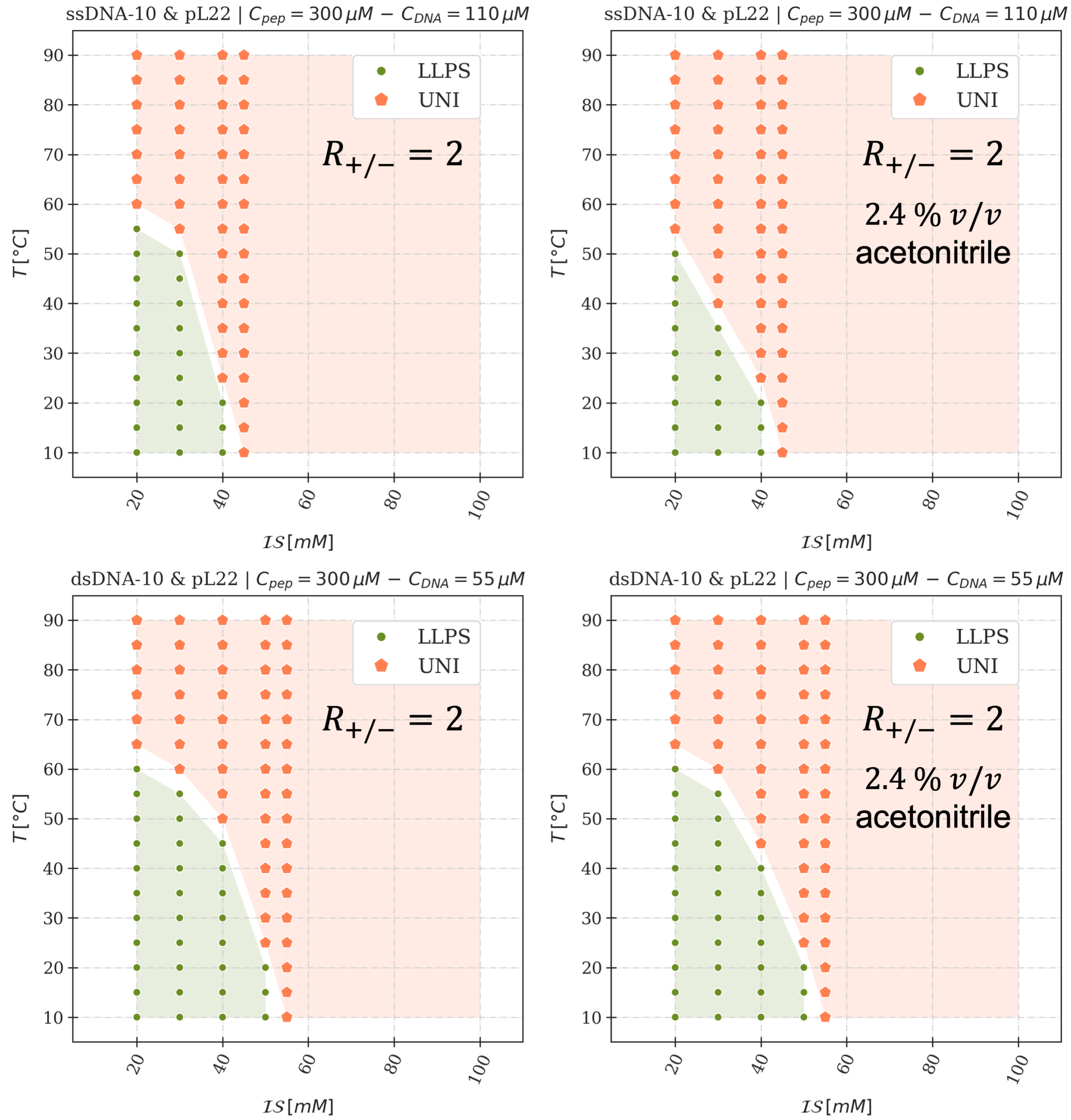

Figure S12: *Experimental phase diagrams of mixtures of pL22 with ssDNA-10 (top row) and dsDNA-10 (bottom row). All phase diagrams refer to mixtures with charge ratio equal to 2 in water solution, 20 mM Tris-HCl pH 7.7 and added NaCl. Samples on the right column contained an additional 2.4% v/v acetonitrile, which was added to obtain the same solvent conditions used for PNA-10 hybrid. Monophasic and biphasic regions are denoted by the color and shape of markers and shaded regions (orange pentagons and green circles, respectively).*

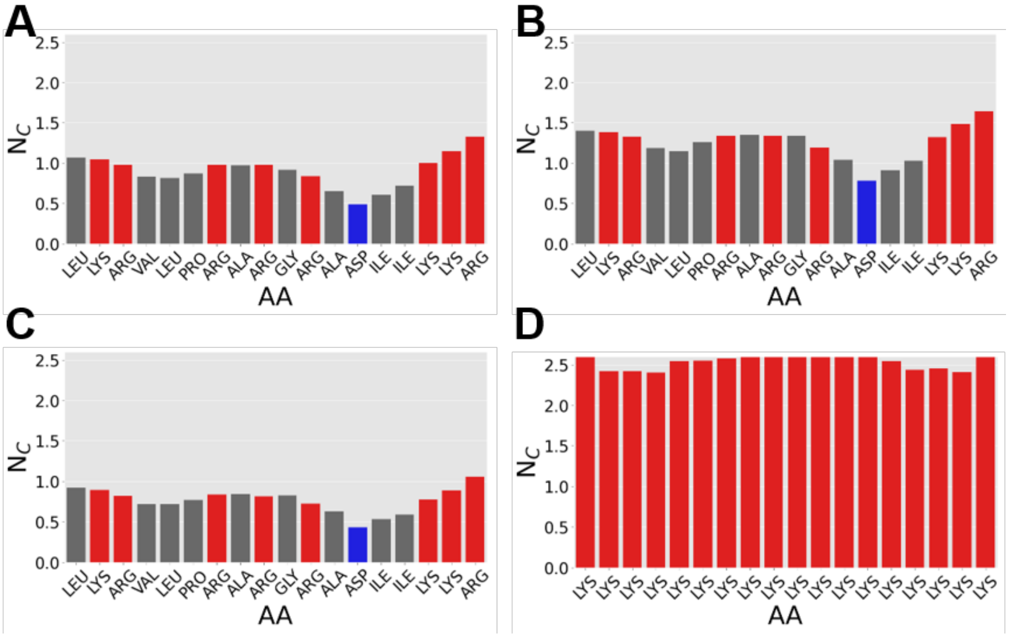

Figure S13: *Average number of contacts with nucleotides, N_c, in simulations at T=303 K for the peptide residues inside the coacervates. A) pL22/ssDNA-20, $\mathcal{IS}$=75 mM (isotropic); B) pL22/dsDNA-20, $\mathcal{IS}$=75 mM (LC); C) pL22/dsDNA-20, $\mathcal{IS}$=225 mM (isotropic); D) polyK-18/dsDNA $\mathcal{IS}$=75 mM (solid).*

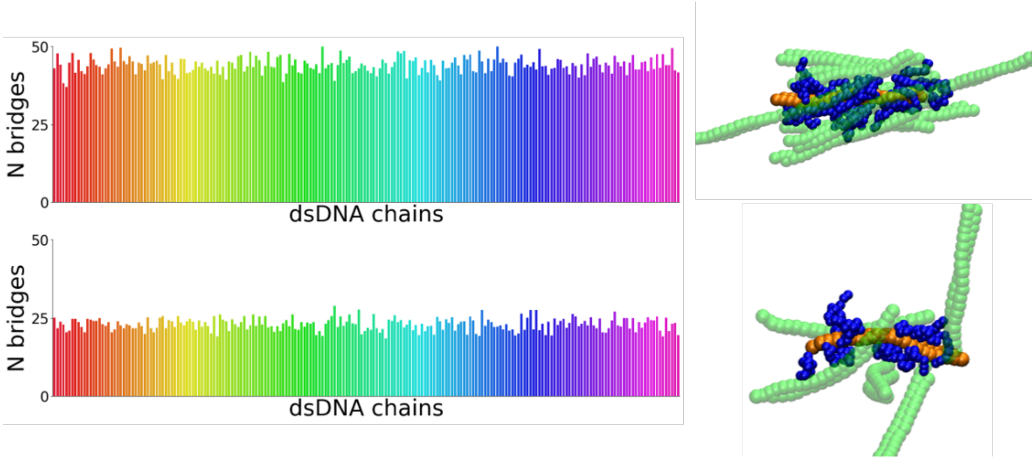

Figure S14: *Average number of inter-DNA contacts bridged by peptide chains within pL22/dsDNA-20 coacervates (barplots), calculated for the individual DNA chains in the sample (from red to violet), and simulation snapshots showing one DNA reference chain (orange) surrounded by other DNA chains (green) bridged to it by peptides (blue). Simulations performed in the LC phase at T=303 K and $\mathcal{IS}$=75 mM (top row), and in the isotropic phase at T=303 K and $\mathcal{IS}$=225 mM (bottom row).*

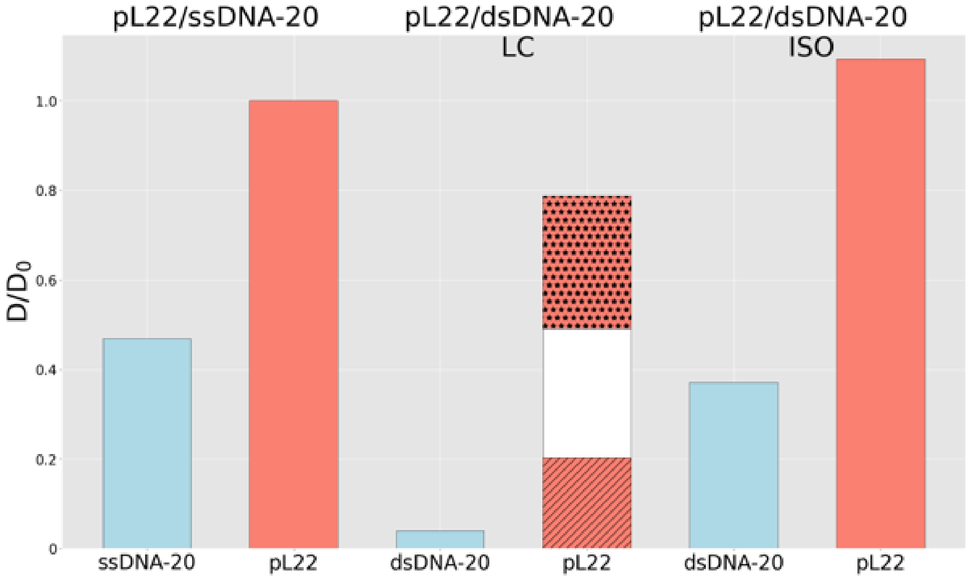

Figure S15: *Translational diffusion coefficient of the center of mass of peptides (orange) and nucleotides (light blue), calculated from MD simulations at T =303 K, for the dense phase of pL22/ssDNA-20 ($\mathcal{IS}$=75 mM) and of pL22/dsDNA-20 (LC at $\mathcal{IS}$=75 and isotropic at $\mathcal{IS}$=225 mM). In the bar for peptides in LC coacervates hatches are used to highlight the perpendicular (lines) and parallel (dots) contribution with respect to the director, while the white color is used to highlight the average diffusion coefficient value.*

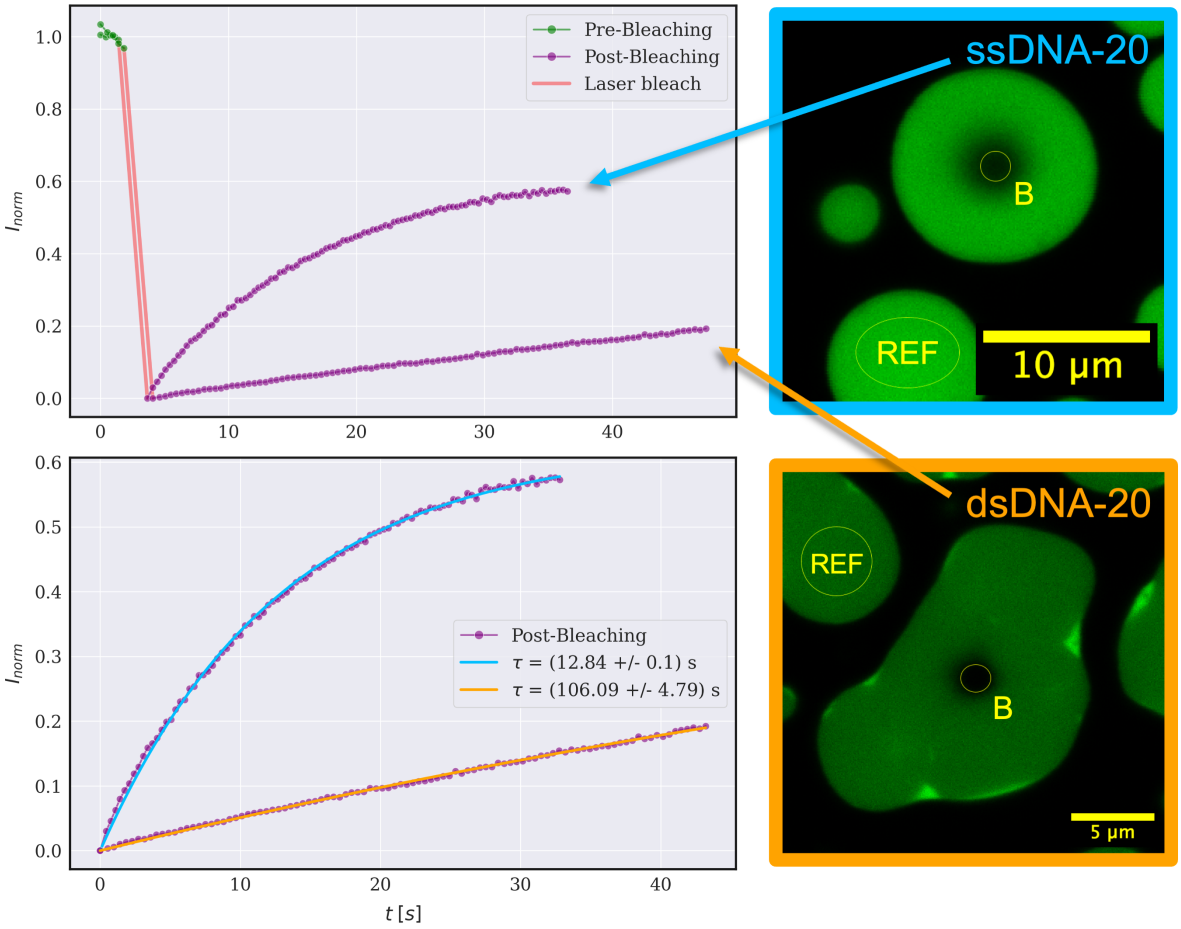

Figure S16: *Fluorescence Recovery After Photobleaching (FRAP) experiment on mixtures of pL22 (C_{pL22}=300 µM) and ssDNA-20 (light blue micrograph, C_{DNA}=110 µM) or dsDNA-20 (orange micrograph, C_{DNA}=55 µM), with 1:200 FAM-labeled DNA strands. The two micrographs show the bleaching spots (yellow circular ROIs, B) and the regions used for photobleaching correction (yellow round ROIs, REF). The recovery curves on top show the normalized fluorescence signals for both samples prior and after laser irradiation, where intensities are processed as described in Material and Methods. The FRAP curve is reproduced on the panel below with the exponential recovery fit for both samples: the estimated characteristic times are* $\tau \sim 13$ *s for ssDNA-20 and* $\tau \sim 106$ *s for dsDNA-20.*

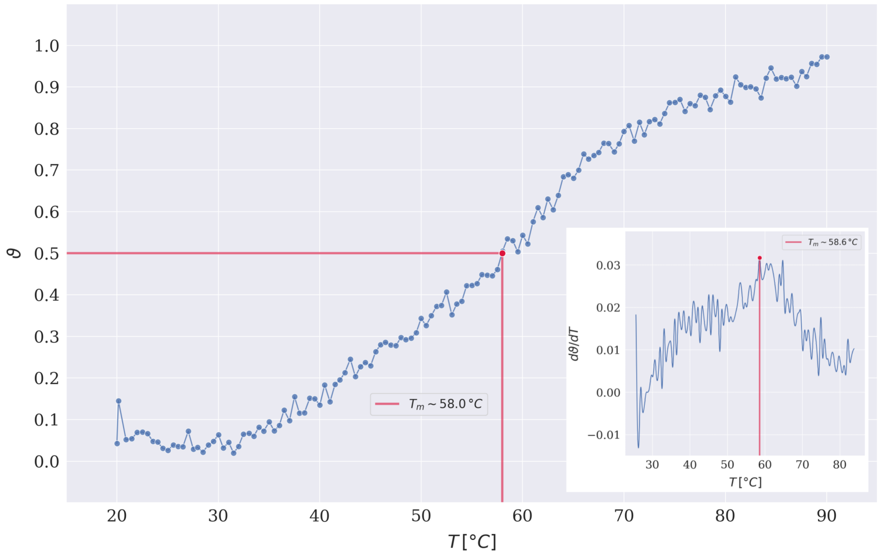

Figure S17: *Normalized absorbance melting curve of PNA-10 hybrid at* $5\,\mu M$ *concentration in water solution, with a small* ($< 1\%$ *v/v*) *amount of acetonitrile. The absorbance spectra in the range 210-320 nm are collected and the raw signals around the 260 nm peak are then averaged and normalized: to a first approximation, the value* ϑ*=0.5 provides the melting temperature (indicated by the red marker and lines), which is found to be around 58°C. The inset of the curve shows the numerical differentiation, upon smoothing and interpolation, with the maximum providing a more reliable estimate of the melting temperature.*

References

[1] Fornace, M. E., Huang, J., Newman, C. T., Porubsky, N. J., Pierce, M. B., and Pierce, N. A. (2022) NUPACK: Analysis and design of nucleic acid structures, devices, and systems. ChemRxiv,.

[2] Dignon, G. L., Zheng, W., Kim, Y. C., Best, R. B., and Mittal, J. (2018) Sequence determinants of protein phase behavior from a coarse-grained model. PLoS Comput. Biol., **14**, e1005941.

[3] Regy, R. M., Dignon, G. L., Zheng, W., Kim, Y. C., and Mittal, J. (2020) Sequence dependent phase separation of protein-polynucleotide mixtures elucidated using molecular simulations. Nucleic Acids Res., **48**(22), 12593–12603.

[4] Regy, R. M., Thompson, J., Kim, Y. C., and Mittal, J. (2021) Improved coarse-grained model for studying sequence dependent phase separation of disordered proteins. Protein Sci., **30**(7), 1371–1379.

[5] Ashbaugh, H. S. and Hatch, H. W. (2008) Natively unfolded protein stability as a coil-to-globule transition in charge/hydropathy space. J. Am. Chem. Soc., **130**, 9536–9542.

[6] Kapcha, L. H. and Rossky, P. J. (2014) A simple atomic-level hydrophobicity scale reveals protein interfacial structure. J. Mol. Biol., **426**, 484–498.

[7] Robertson, M. J., Qian, Y., Robinson, M. C., Tirado-Rives, J., and Jorgensen, W. L. (2019) Development and testing of the OPLS-AA/M force field for RNA. J. Chem. Theory Comput., **15**(4), 2734–2742.

[8] Malhotra, I. and Potoyan, D. A. (2023) Re-entrant transitions of locally stiff RNA chains in the presence of polycations leads to gelated architectures. Soft Matter, **19**, 5622–5629.

[9] Malmberg, C. G. and Maryott, A. A. (1956) Dielectric Constant of Water from 00 to 1000 C. J. Res. Natl. Bur. Stand., **56**(1), 1–8.

[10] Neidle, S. (2008) Principles of Nucleic Acid Structure, Academic Press, New York, .

[11] Smith, S., Cui, Y., and Bustamante, C. (1996) Overstretching B-DNA: The elastic response of individual duble-stranded and single-stranded DNA. Science, **271**, 795–799.

[12] Yu, B., Liang, H., Rumyantsev, A. M., and de Pablo, J. J. (2022) Isotropic-to-nematic transition is salt-free polyeletrolyte coacervates from coarse-grained simulations. Macromolecules, **55**, 9627–9639.

[13] Thompson, A. P., Aktulga, H. M., Berger, R., Bolintineanu, D. S., Brown, W. M., Crozier, P. S., in't Veld, P. J., Kohlmeyer, A., Moore, S. G., Nguyen, T. D., Shan, R., Stevens, M. J., Tranchid, J., Trott, C., and Plimpton, S. J. (2022) LAMMPS-a flexible simulation tool for particle-based materials modeling at the atomic, meso, and continuum scales. Comput. Phys. Commun., **271**, 108171.

[14] Schneider, T. and Stoll, E. (1978) Molecular-dynamics study of a three-dimensional one-component model for distortive phase transitions. Phys. Rev. B, **17**, 1302–1322.

[15] Hoover, W. G. (1986) Constant-pressure equations of motion. Phys. Rev. A, **A34**, 2499–2500.

[16] Gowers, R. J., Linke, M., Barnoud, J., Reddy, T. J. E., Melo, M. N., Seyler, S. L., Domanski, J., Dotson, D. L., Buchoux, S., Kenney, I. M., , and Beckstein, O. (2019) MDAnalysis: a Python package for the rapid analysis of molecular dynamics simulations. In Benthall, S. and Rostrup, S., (eds.), Proceedings of the 15th Python in Science Conference, Austin, TX: pp. 98–105.

[17] Luckhurst, G. R. and Gray, G. W. (1979) The Molecular Physics of Liquid Crystals, Academic Press, New York, .

[18] Snodin, B. E., Randisi, F., Mosayebi, M., Šulc, P., Schreck, J. S., Romano, F., Ouldridge, T. E., Tsukanov, R., Nir, E., Louis, A. A., et al. (2015) Introducing improved structural properties and salt dependence into a coarse-grained model of DNA. J. Chem. Phys., **142**(23).

[19] Poppleton, E., Bohlin, J., Matthies, M., Sharma, S., Zhang, F., and Šulc, P. (2020) Design, optimization and analysis of large DNA and RNA nanostructures through interactive visualization, editing and molecular simulation. Nucleic Acids Res., **48**, e72.

[20] Ouldridge, T. E., Louis, A. A., and Doye, J. P. K. (2011) Structural, mechanical, and thermodynamic properties of a coarse-grained DNA model. J. Chem. Phys., **134**, 085101.

[21] Jorgensen, W. L., Chandrasekhar, J., Madura, J. D., Impey, R. W., and Klein, M. L. (1983) Comparison of simple potential functions for simulating liquid water. J. Chem. Phys., **79**(2), 926–935.

[22] Jorgensen, W. L. and Tirado-Rives, J. (1988) The OPLS force field for proteins. Energy minimizations for crystals of cyclic peptides and crambin. J. Am. Chem. Soc., **110**(6), 1657–1666.

[23] Jurrus, E., Engel, D., Star, K., Monson, K. ., Brandi, J., Felberg, L. E., Brookes, D. H., Wilson, L., Chen, J., Liles, K., Chun, M., Li, P., Gohara, D. W., Dolinsky, T., Konecny, R., Koes D. R., Nielsen, J. E., Head-Gordon, T., Geng, W., Krasny, R., Wei, G. W., Holst, M. J., McCammon, J. A., and Baker, N. A. (2018) Improvements to the APBS biomolecular solvation software suite. Protein Sci., **27**, 112–128.

[24] Georgiev, D., Pedersen, S. V., Xie, R., Fernandez-Galiana, A., Stevens, M. M., and Barahona, M. (2024) RamanSPy: An Open-Source Python Package for Integrative Raman Spectroscopy Data Analysis. Anal. Chem., **96**, 8492–8500.
